# p113 isoform encoded by *CUX1* circular RNA drives tumor progression via facilitating ZRF1/BRD4 transactivation

**DOI:** 10.1186/s12943-021-01421-8

**Published:** 2021-09-27

**Authors:** Feng Yang, Anpei Hu, Yanhua Guo, Jianqun Wang, Dan Li, Xiaojing Wang, Shikai Jin, Boling Yuan, Shuang Cai, Yi Zhou, Qilan Li, Guo Chen, Haiyang Gao, Liduan Zheng, Qiangsong Tong

**Affiliations:** 1 Department of Pediatric Surgery, Union Hospital, Tongji Medical College, Huazhong University of Science and Technology, 1277 Jiefang Avenue, Wuhan, Hubei Province 430022 People’s Republic of China; 2 Clinical Center of Human Genomic Research, Union Hospital, Tongji Medical College, Huazhong University of Science and Technology, 1277 Jiefang Avenue, Wuhan, Hubei Province 430022 People’s Republic of China; 3 Department of Geriatrics, Union Hospital, Tongji Medical College, Huazhong University of Science and Technology , 1277 Jiefang Avenue, Wuhan, Hubei Province 430022 People’s Republic of China; 4 Department of Pathology, Union Hospital, Tongji Medical College, Huazhong University of Science and Technology, 277 Jiefang Avenue, Wuhan, Hubei Province 430022 People’s Republic of China; 5 Department of Gastrointestinal Surgery, Union Hospital, Tongji Medical College, Huazhong University of Science and Technology, 1277 Jiefang Avenue, Wuhan, Hubei Province 430022 People’s Republic of China

**Keywords:** Circular RNA-coding protein, Zuotin-related factor 1, Bromodomain protein 4, Neuroblastoma progression

## Abstract

**Background:**

Metabolic reprogramming sustains tumorigenesis and aggressiveness of neuroblastoma (NB), the most common extracranial malignancy in childhood, while underlying mechanisms and therapeutic approaches still remain elusive.

**Methods:**

Circular RNAs (circRNAs) were validated by Sanger sequencing. Co-immunoprecipitation, mass spectrometry, chromatin immunoprecipitation (ChIP) sequencing, and RNA sequencing assays were applied to explore protein interaction and target genes. Gene expression regulation was observed by ChIP, dual-luciferase reporter, real-time quantitative RT-PCR, and western blot assays. Gain- and loss-of-function studies were performed to observe the impacts of circRNA-encoded protein and its partners on the lipid metabolism, mitochondrial activity, growth, invasion, and metastasis of NB cells.

**Results:**

A novel 113-amino acid protein (p113) of CUT-like homeobox 1 (*CUX1*) was identified in NB cells treated by serum deprivation. Further validating studies revealed that nuclear p113 was encoded by circRNA of *CUX1*, and promoted the lipid metabolic reprogramming, mitochondrial activity, proliferation, invasion, and metastasis of NB cells*.* Mechanistically, p113 interacted with Zuotin-related factor 1 (ZRF1) and bromodomain protein 4 (BRD4) to form a transcriptional regulatory complex, and mediated the transactivation of ZRF1/BRD4 in upregulating *ALDH3A1*, *NDUFA1*, and *NDUFAF5* essential for conversion of fatty aldehydes into fatty acids, fatty acid β-oxidation, and mitochondrial complex I activity. Administration of an inhibitory peptide blocking p113-ZRF1 interaction suppressed the tumorigenesis and aggressiveness of NB cells. In clinical NB cases, high expression of *p113*, *ZRF1*, or *BRD4* was associated with poor survival of patients.

**Conclusions:**

These results indicate that p113 isoform encoded by *CUX1* circular RNA drives tumor progression via facilitating ZRF1/BRD4 transactivation.

**Supplementary Information:**

The online version contains supplementary material available at 10.1186/s12943-021-01421-8.

## Background

Neuroblastoma (NB), a solid malignancy featured by rapid progression and high mortality, accounts for more than 15% of tumor-related deaths in pediatric population [1]. For high-risk NB patients, the clinical outcome still remains unfavorable in despite of multimodal therapeutics [1]. Beside aerobic glycolysis and glutaminolysis, tumor cells obtain energies from lipid metabolism for thriving in challenging environments, including uptake of fatty acids, de novo lipid synthesis [2], and fatty acid β-oxidation (FAO) [3]. FAO allows for mitochondrial conversion of long-chain fatty acids into acetyl-CoA, which is subsequently oxidized via tricarboxylic acid cycle and electron transport chain (ETC) to produce ATP [4]. FAO pathway is dysregulated in diverse human malignancies [3], and tumor cells rely on FAO for proliferation, survival, stemness, drug resistance, or metastasis [5]. However, transcriptional regulators of lipid metabolic reprogramming in NB still remain largely elusive.

Circular RNAs (circRNAs) are a subclass of non-coding RNAs (ncRNAs) with closed continuous loops, and act as key regulators of gene expression in cancers by serving as miRNA (microRNA) sponges or RNA-binding protein partners [6]. For example, *CDR1as* exerts miRNA sponging activity as a competing endogenous RNA [7]. Eukaryotic translation elongation factor 3 J (*EIF3J*)-derived circRNA (*circ-EIF3J*) interacts with U1 snRNP and RNA polymerase II to facilitate parental gene transcription [8]. *Circ-AMOTL1* derived from angiomotin-like 1 (*AMOTL1*) promotes tumorigenesis by increasing nuclear retention of c-Myc [9]. Meanwhile, some circRNAs have the capability to encode peptides or proteins [10–13]. For example, circRNA-coding protein FBXW7-185aa inhibits glioblastoma proliferation by reducing c-Myc stabilization [10]. A novel protein encoded by circular RNA of AKT serine/threonine kinase 3 (*circAKT3*) suppresses glioblastoma tumorigenesis by competing with active phosphoinositide-dependent kinase 1 [12]. Meanwhile, β-catenin circular RNA (*circβ-catenin*)-derived protein promotes growth of liver cancer cells via activating Wnt pathway [11]. Circular RNA of protein phosphatase 1 regulatory subunit 12A (*circPPP1R12A*) encodes a protein that drives metastasis of colon cancer via Hippo-YAP signaling pathway [13]. However, the roles and underlying mechanisms of circRNA-coding proteins in NB remain to be determined.

In this study, we identify a 113-amino acid protein (p113) encoded by circRNA of CUT-like homeobox 1 (*CUX1*) as a driver of NB progression. Mechanistically, p113 interacts with Zuotin-related factor 1 (ZRF1) and bromodomain protein 4 (BRD4) to form a transcriptional regulatory complex, resulting in upregulation of aldehyde dehydrogenase 3 family member A1 (*ALDH3A1*), NADH:ubiquinone oxidoreductase subunit A1 (*NDUFA1*), and NADH: ubiquinone oxidoreductase complex assembly factor 5 (*NDUFAF5*) essential for conversion of fatty aldehydes into fatty acids, FAO, and mitochondrial complex I activity for ATP production. Administration of an inhibitory peptide blocking p113-ZRF1 interaction suppresses FAO, mitochondrial complex I activity, tumorigenesis, and aggressiveness of NB cells, indicating the oncogenic roles of *p113* and *ZRF1* in lipid metabolic reprogramming and NB progression.

## Materials and methods

### Cell culture

Human NB cell lines SH-SY5Y (CRL-2266), SK-N-SH (HTB-11), SK-N-BE(2) (CRL-2271), BE(2)-C (CRL-2268), and IMR-32 (CCL-127), cervical cancer HeLa (CCL-2) cells, prostate cancer PC-3 cells (CRL-1435), nontransformed mammary epithelial MCF 10A (CRL-10317), and embryonic kidney HEK293T (CRL-3216) were obtained from American Type Culture Collection (Rockville, MD). Cell lines were authenticated by short tandem repeat profiling, and used within 6 months after resuscitation. Mycoplasma was regularly examined with Lookout Mycoplasma PCR Detection Kit (Sigma, St. Louis, MO). Tumor cells and HEK293T cells were cultured in RPMI1640 supplied with 10% fetal bovine serum (Gibco, Grand Island, NY), while MCF 10A cells were cultured in DMEM/F12 medium containing 5% horse serum (Invitrogen, Carlsbad, CA) and 20 ng/ml epidermal growth factor (Peprotech, Rocky Hill, NJ). For metabolic SD stress, cells were maintained in RPMI1640 containing 1% fetal bovine serum.

### RT-PCR and real-time quantitative PCR

Nuclear, cytoplasmic, and total RNAs were extracted using RNA Subcellular Isolation Kit (Active Motif, Carlsbad, CA) or RNeasy Mini Kit (Qiagen Inc., Redwood City, CA). For circRNA detection, RNase R (3 U/ug, Epicenter, Madison, WI) treatment was undertaken at 37 °C for 15 min, while reverse transcription kit (TakaRa, Dalian, China) was used for cDNA synthesis. Quantification of mRNA and circRNA was performed using a SYBR Green PCR Master Mix (Applied Biosystems, Carlsbad, CA) and primers (Additional file 1: Table S1).

### Western blot assay

Proteins were extracted with RIPA lysis buffer (Thermo Fisher Scientific, Inc., Waltham, MA). Anti-p113 polyclonal antibody was prepared by immunizing rabbits with synthesized peptide corresponding to C-terminus of p113 (EQQLSAKNSTLKGRRD; ABclonal Biotechnology Co., Ltd., Wuhan, China). Western blot analysis was performed as previously described [14–16], with antibodies specific for CUX1 (ab230844), transcription factor 3 (TCF3, ab69999), ALDH3A1 (ab186726), NDUFA1 (ab249923), NDUFAF5 (ab240971), β-actin (ab179467, Abcam Inc., Cambridge, MA), CUX1 (sc-514,008), histone H3 (sc-517,576), glyceraldehyde 3-phosphate dehydrogenase (GAPDH, sc-47724), glutathione S-transferase (GST, sc-33614, Santa Cruz Biotechnology, Santa Cruz, CA), Flag-tag (14793S), ZRF1 (12844S), BRD4 (13440S), hemagglutinin (HA)-tag (3724S), or His-tag (12698S, Cell Signaling Technology Inc., Danvers, MA).

### Plasmid construction and stable transfection

Linear *ecircCUX1* (*hsa_circ_30402*) was obtained from NB tissues by PCR (Additional file 1: Table S2) and inserted into pLCDH-ciR (Geenseed Biotech Co., Guangzhou, China). The *ecircCUX1*-3Flag construct was established by inserting a 3 × Flag-coding sequence upstream of TGA codon within linear *ecircCUX1* (Additional file 1: Table S2). Mutation of *ecircCUX1* was performed with GeneTailor™ Site-Directed Mutagenesis System (Invitrogen) and primers (Additional file 1: Table S2). Synthesized p113-3Flag sequence was subcloned into pcDNA3.1-mini (Addgene, Cambridge, MA), while p113-3Flag with circular frames was ligated into CV186 (Genechem Co., Ltd., Shanghai, China) or pCMV-HA (Addgene). Human *ZRF1* cDNA (1866 bp) was obtained from Genechem Co., Ltd., while *BRD4* cDNA (4089 bp) was provided by Dr. Guosong Jiang [17]. Truncations of *ZRF1* or *BRD4* were obtained by PCR amplification (Additional file 1: Table S2) and subcloned into pCMV-3Tag-1A (Stratagene, Santa Clara, CA), pCDNA4-His (Invitrogen), pGEX-6P-1 (Addgene), or pMal-c4X (Addgene), respectively. The pGEX-6P-1 or pMal-c4X constructs were transformed into *E. coli* to produce GST-tagged ZRF1 or maltose-binding protein (MBP)-tagged BRD4 proteins. Single guide RNAs (sgRNAs) targeting downstream region of gene transcription start site (Additional file 1: Table S3) were inserted into dCas9-BFP-KRAB (Addgene) [18]. Oligonucleotides specific for short hairpin RNAs (shRNAs, Additional file 1: Table S3) were inserted into GV298 (Genechem Co., Ltd). Lentiviral plasmids were co-transfected with psPAX2 and pMD2G into HEK293T cells to generate infectious lentivirus. Stable cell lines were obtained with puromycin selection for 3-4 weeks.

### Dual-luciferase reporter assay

The internal ribosome entry site (IRES) reporter of *ecircCUX1* was amplified with primers (Additional file 1: Table S2) and subcloned into pGL3-Basic (Promega, Madison, WI). Human ZRF1 activity reporter was established by inserting oligonucleotides containing four canonical binding sites (Additional file 1: Table S2) into pGL3-Basic (Promega). Promoter fragment of *ALDH3A1* (1179 bp), *NDUFA1* (1077 bp), or *NDUFAF5* (1079 bp) was amplified from genomic DNA with primers (Additional file 1: Table S2) and subcloned into pGL3-Basic (Promega). Dual-luciferase assay was performed according to the manufacturer’s instructions (Promega).

### Knockin with CRISPR-Cas9 genome editing

Genomic insertion of a 3 × Flag coding sequence or mutation of *ecircCUX1* was performed by homology-directed repair of a CRISPR-Cas9-induced DNA break. The sgRNA oligonucleotides targeting exon 9 of *CUX1* were designed using online program (http://crispr.mit.edu), and ligated into LentiCRISPR V2 vector (Addgene). Donor DNA carrying 1200 bp of homologous sequences upstream and downstream exon 9 of *CUX1* with 3 × Flag insertion or open reading frame (ORF) mutation was synthesized. Cells were co-transfected with sgRNA and donor DNA, selected with puromycin for 2-3 weeks, and validated by PCR amplification using primers (Additional file 1: Table S1) and Sanger sequencing.

### RNA sequencing (RNA-seq)

Total RNA of tumor cells (1 × 10^6^) was isolated using TRIzol™ reagent (Life Technologies, Inc., Gaithersburg, MD). Library preparation and transcriptome sequencing on a BGIseq500 platform were carried out at Beijing Genomics Institute (BGI-Shenzhen, China).

### Co-immunoprecipitation and mass spectrometry

Co-immunoprecipitation (co-IP) was performed as previously described [14–16], with antibodies for p113 (ABclonal Biotechnology Co., Ltd), GST-tag (sc-33614, Santa Cruz Biotechnology), Flag-tag (14793S), ZRF1 (12844S), BRD4 (13440S), HA-tag (3724S), His-tag (12698S), MBP-tag (2396S, Cell Signaling Technology Inc., MA). Bead-bound proteins were analyzed by Coomassie blue staining, western blot, or mass spectrometry (Wuhan Institute of Biotechnology, Wuhan, China).

### Chromatin immunoprecipitation (ChIP) and ChIP sequencing (ChIP-seq)

ChIP assay was undertaken using EZ-ChIP kit (Upstate Biotechnology, Temacula, CA) [14–16], with antibodies specific for p113 (ABclonal Biotechnology Co., Ltd), ZRF1 (12844S), or BRD4 (13,440, Cell Signaling Technology Inc.) and primers (Additional file 1: Table S1). ChIP-seq was undertaken at Wuhan Seqhealth Technology Co., Ltd. (Wuhan, China).

### Immunofluorescence staining

Cells were seeded on coverslips and incubated with antibody specific for p113 (ABclonal Biotechnology Co., Ltd), Flag-tag (14793S, Cell Signaling Technology Inc.), 4-hydroxynonenal (4-HNE, MAB3249, Novus Biologicals, Ltd., Centennial, CO), or ALDH3A1 (ab186726, Abcam Inc.) at room temperature for 2 h. Then, coverslips were incubated with Alexa Fluor 594 goat anti-mouse IgG or Alexa Fluor 488 goat anti-rabbit IgG, and stained with 4′,6-diamidino-2-phenylindole (DAPI, 300 nmol·L^− 1^, Sigma).

### Bimolecular fluorescence complementation (BiFC) assay

Human *p113* ORF (342 bp), *ZRF1* cDNA (1866 bp), and *BRD4* cDNA (4089 bp) were subcloned into pBiFC-VN173 or pBiFC-VC155 (Addgene), and co-transfected into tumor cells with Lipofectamine 2000 (Invitrogen) for 24 h. The fluorescence was observed with a confocal microscope (Nikon, Japan) [15, 16, 19].

### Design and synthesis of inhibitory peptides

Inhibitory peptides for blocking interaction between p113 and ZRF1 were designed. The 11-amino acid long peptide (YGRKKRRQRRR) from Tat protein transduction domain served as a cell-penetrating peptide. Thus, inhibitory peptides were chemically synthesized by linking with N-terminal biotin-labeled cell-penetrating peptide and C-terminal fluorescein isothiocyanate (FITC, ChinaPeptides Co. Ltd., Shanghai, China), with purity larger than 95%.

### Biotin-labeled peptide pull-down assay

Cellular proteins were isolated using 1× cell lysis buffer (Promega), and incubated with biotin-labeled peptide and streptavidin-agarose at 4 °C for 2 h. Beads were extensively washed, and protein pulled down was measured by western blot.

### Sucrose gradient sedimentation

Sedimentation of polysomal fractions was performed as previously described [20]. Briefly, tumor cells were treated with 100 μg/ml of cycloheximide (Sigma) for 5-10 min. Cell extracts were layered on top of 15-30% (w/v) linear sucrose gradient and centrifugated (40,000×g) at 4 °C for 2 h. Polysome-bound transcripts in collected gradient fractions were detected by real-time qRT-PCR.

### Lipid profiling assay

Target metabolic profiling was performed by gas chromatography-mass spectrometry (GC-MS) at Wuhan Metware Biotechnology Co, Ltd. (Wuhan, China). Briefly, samples were quickly frozen in liquid nitrogen, ultrasonically crushed, and filtered through SPE column. Then, SPE column was extracted with n-hexane and detected by GC-MS.

### Seahorse extracellular flux assay

Cells were seeded in XF cell culture microplates (30,000/well) at 37 °C for 24 h, cultured in FAO assay medium (pH 7.4, 2.5 mmol·L^− 1^ glucose, 0.5 mmol·L^− 1^ carnitine) without CO_2_ for 1 h, and incubated with bovine serum albumin (BSA) or oleic acid-BSA (200 μmol·L^− 1^, Sigma). Then, oligomycin (1 μmol·L^− 1^), carbonyl cyanide-p-trifluoromethoxyphenylhydrazone (FCCP, 2 μmol·L^− 1^), and rotenone/antimycin A (0.5 μmol·L^− 1^, Sigma) were added, while oxygen consumption rate (OCR) was detected using a Seahorse Biosciences XFe24 Flux Analyzer (North Billerica, MA).

### Mitochondrial structure, membrane potential (Δψm), and complex I activity assays

For imaging mitochondrial mass or morphology independent of membrane potential [21], live cells were stained by MitoTracker Green (100 nmol·L^− 1^, Invitrogen) at 37 °C for 30 min or transfected with MitoRFP reporter (Addgene) for 48 h, with nuclei staining by Hochest 33,342 (Sigma). Mitochondrial structure was observed by transmission electron microscopy [22]. Cells were treated with JC-1 (10 μg/ml, Sigma) at 37 °C for 20 min, while red fluorescence intensity reflecting Δψm was assessed with a fluorescence microscope. Mitochondrial complex I activity was determined by Mitochondrial Complex I Activity Assay Kit (Sigma).

### NAD^+^/NADH ratio and ATP level measurement

Cellular nicotinamide adenine dinucleotide (NAD^+^)/nicotinamide adenine dinucleotide (NADH) ratio or ATP levels were measured using NAD^+^/NADH Assay Kit (ab65348, Abcam Inc.) and ATP Determination Kit (A22066, Invitrogen), respectively.

### In vitro cell viability, growth, and invasion assays

In vitro viability, growth, and invasive capabilities of tumor cells were detected by 2-(4,5-dimethyltriazol-2-yl)-2,5-diphenyl tetrazolium bromide (MTT; Sigma) colorimetric [23, 24], soft agar [15, 16, 19], and matrigel invasion [15, 16, 19] assays.

### In vivo tumorigenesis and aggressiveness assays

Four-week-old male BALB/c nude mice (National Rodent Seeds Center, Shanghai, China) were reared at specific pathogen free condition. For in vivo tumor growth, metastasis, and peptide therapeutic studies, tumor cells (1 × 10^6^ or 0.4 × 10^6^) were injected into dorsal flanks or tail vein of blindly randomized nude mice (*n* = 5 per group), respectively. For therapeutic studies, 1 week after injection, mice were blindly randomized and treated by tail vein injection of synthesized cell-penetrating peptides (ChinaPeptides, Shanghai, China) as indicated [15, 16, 19].

### Human tissue samples

All procedures were conformed to principles set forth by Declaration of Helsinki. Written informed consent was obtained from all legal guardians of patients without a history of preoperative chemotherapy or radiotherapy. Human normal dorsal root ganglia were collected from therapeutic abortion. Fresh tumor tissues were collected at surgery, validated by pathological diagnosis, and stored at − 80 °C.

### Immunohistochemistry

Immunohistochemical staining and quantitative evaluation were performed as previously described [15, 16, 19], with antibody specific for p113 (ABclonal Biotechnology Co.), Ki-67 (ab92742, Abcam Inc.), or CD31 (ab28364, Abcam Inc.).

### Data and code availability

RNA-seq and ChIP-seq data have been deposited in Gene Expression Omnibus (GEO) database (https://www.ncbi.nlm.nih.gov/geo/, accession number **GSE182329 and GSE182402)**. Public datasets are available from GEO database (GSE62564, GSE25066, GSE17679, GSE65904, GSE2658) or The Cancer Genome Atlas (TCGA) database (https://cancergenome.nih.gov).

### Statistical analysis

All data were shown as mean ± standard error of the mean (s.e.m.). Cutoff values were determined by average gene expression levels. Student’s *t* test, analysis of variance (ANOVA), and χ^2^ analysis were applied to compare difference. Fisher’s exact test was applied to analyze statistical significance of overlapping. Log-rank test was used to assess survival difference. All statistical tests were two-sided and considered significant when false discovery rate (FDR)-corrected *P* values were less than 0.05.

## Results

### Serum deprivation stress promotes expression of p113 derived from a circRNA of *CUX1* in NB

**Fig. 1:**
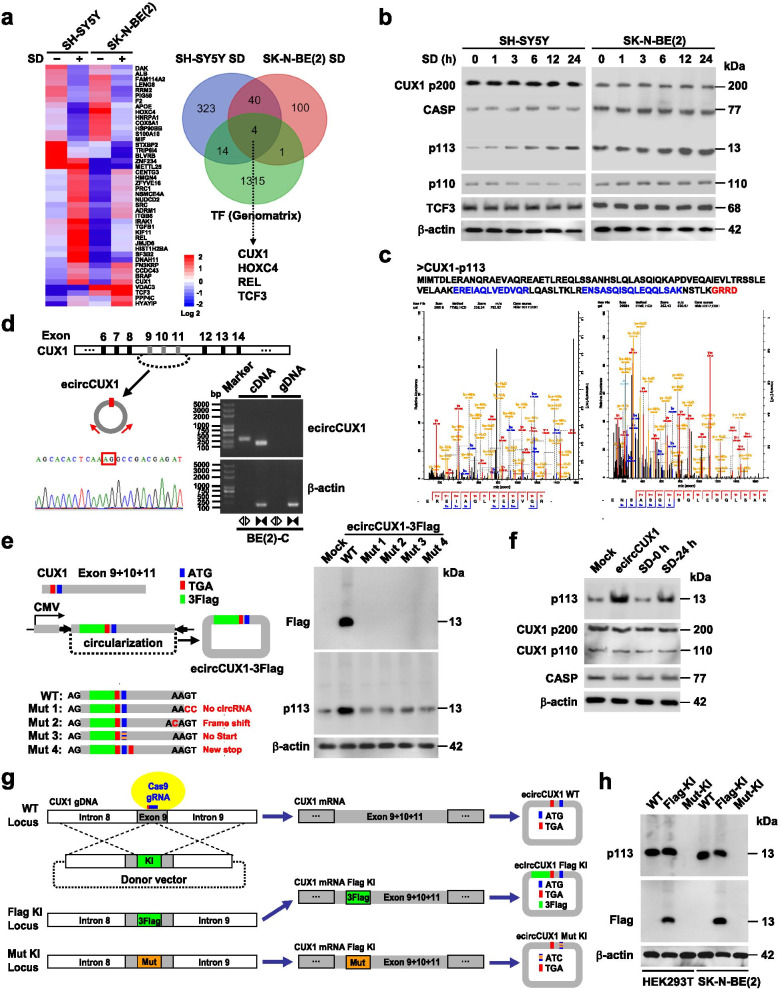
Serum deprivation stress promotes expression of p113 derived from a circRNA of *CUX1* in NB. a Heatmap of proteomics (left panel) and Venn diagram (right panel) showing altered proteins in SH-SY5Y and SK-N-BE(2) cells treated by serum deprivation (SD) for 24 h, and overlapping analysis with established transcription factors derived from Genomatrix database (http://www.genomatix.de). **b** Western blot assay indicating the expression of CUX1 isoforms and TCF3 in SH-SY5Y and SK-N-BE(2) treated with SD as indicated. **c** Mass spectrometry assay revealing amino acid sequence of p113 recovered from electrophoresis gel, with additional amino acids produced by *ecircCUX1* (red). **d** Schematic illustration showing the genomic location of *ecircCUX1* generated from exons 9-11 of *CUX1*, and validation by RT-PCR using convergent or divergent primers and Sanger sequencing. **e** Western blot assay (right panel) indicating endogenous and exogenous expression of p113 in SH-SY5Y cells stably transfected with empty vector (mock), wild-type or mutant 3Flag-tagged *ecircCUX1* as indicated (left panel). **f** Western blot assay showing the levels of p113 and CUX1 isoforms in SH-SY5Y cells stably transfected with mock or *ecircCUX1*, and those treated with or without SD for 24 h. **g** Schematic illustration indicating genomic knockin (KI) of 3Flag-tagged or mutant (Mut) form of *ecircCUX1* using CRISPR-Cas9. **h** Western blot assay revealing endogenous and exogenous expression of p113 in HEK293T and SK-N-BE(2) cells with genomic knockin (KI) of 3Flag-tagged or mutant (Mut) form of *ecircCUX1*. Fisher’s exact test for overlapping analysis in **a**. Data are representative of three independent experiments in **b**, **d**-**f** and **h**

To investigate crucial factors regulating metabolic reprogramming, NB cell lines were treated by serum deprivation (SD), a condition causing metabolic stress [25], while altered proteins were analyzed by isobaric tags for relative and absolute quantification (iTRAQ). There were 381 and 145 differentially expressed proteins in SH-SY5Y and SK-N-BE(2) cells upon SD for 24 h, respectively (Additional file 1: Table S4), and overlapping with Genomatix database (http://www.genomatix.de/) indicated that expression of four transcription factors was significantly altered (Fig. 1a). Among them, CUT-like homeobox 1 (CUX1) and TCF3 were consistently upregulated in both NB cell lines. Validating western blot assay indicated that expression of major CUX1 isoforms [p200, p110, or CDP/CUT alternatively spliced product (CASP)] and TCF3 remained unaffected, while a new CUX1 isoform with a molecular weight of 13 kDa was unexpectedly upregulated in a time-dependent manner upon SD treatment (Fig. 1b). The amino acid sequence of this new isoform was deduced from exons 10 and 11 of *CUX1* (Fig. 1c). Analysis from circRNADb database (http://reprod.njmu.edu.cn/circrnadb) identified a spanning junction ORF-containing circRNA (*hsa_circ_30402*) derived from exons 9-11 of *CUX1* (referred as *ecircCUX1*), which might encode a conserved 113-amino acid protein (p113) in primates (Fig. 1d and Additional file 1: Fig. S1a). Accurate circularization of *ecircCUX1* was validated in BE(2)-C cells (Fig. 1a), with cytoplasmic abundance (Additional file 1: Fig. S1b). Notably, SD treatment did not affect the *ecircCUX1* levels (Additional file 1: Fig. S1c), but increased their binding to polysomes in NB cells (Additional file 1: Fig. S1d). Using prepared p113 antibody or Flag-tag antibody, western blot assay revealed the increase of p113 levels in NB cells transfected with Flag-tagged wild-type *ecircCUX1*, but not with ORF mutant vector (Fig. 1e). In addition, ectopic expression of *ecircCUX1* or SD treatment led to upregulation of p113, but not of p200, p110 or CASP, in NB cells (Fig. 1f). Next, CRISPR-Cas9-mediated knockin of Flag-tagged *ecircCUX1*, but not of mutant *ecircCUX1*, resulted in p113 expression in HEK293T and SK-N-BE(2) cells (Fig. 1g, h and Additional file 1: Fig. S1e, f). Importantly, IRES of *ecircCUX1* induced a remarkable luciferase activity (Additional file 1: Fig. S1g), indicating its 5′ cap-independent translation pattern. These results demonstrated that SD stress promoted the expression of p113 derived from a circRNA of *CUX1* in NB cells.

### p113 is upregulated and facilitates fatty acid oxidation and mitochondrial activity in NB

**Fig. 2:**
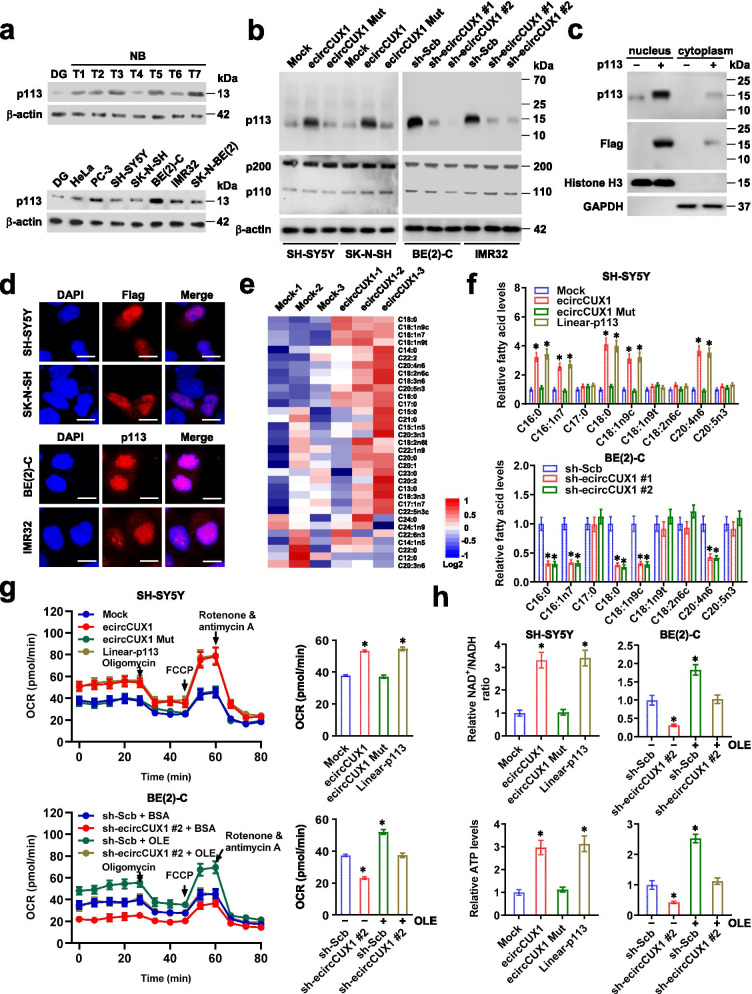
p113 is upregulated and facilitates fatty acid oxidation and mitochondrial activity in NB. **a** Western blot assay showing the p113 levels in normal dorsal ganglia (DG), tumor (T) tissues of NB, and cultured tumor cell lines. **b** Western blot assay indicating the expression of p113 and CUX1 isoforms (p200 and p110) in SH-SY5Y, SH-N-SH, BE(2)-C, and IMR32 cells stably transfected with empty vector (mock), *ecircCUX1*, *ecircCUX1* with ORF mutation (*ecircCUX1* Mut), scramble shRNA (sh-Scb), or sh-ecircCUX1. **c** Western blot assay showing the cytoplasmic and nuclear accumulation of p113 in SH-SY5Y cells stably transfected with mock or *p113*. **d** Immunofluorescence assay revealing cytoplasmic and nuclear localization of p113 in SH-SY5Y and SK-N-SH cells stably transfected with Flag-tagged p113 (upper panel), and those in BE(2)-C and IMR32 cells (lower panel), with nuclei stained by DAPI (blue). Scale bar: 10 μm. **e** and **f** Heatmap (e) and quantification (f) of metabolite profiling assay indicating the fatty acid levels in SH-SY5Y and BE(2)-C cells stably transfected with mock, *ecircCUX1*, *ecircCUX1* Mut, *p113*, sh-Scb, or sh-ecircCUX1 (*n* = 3). **g** Seahorse extracellular flux assay showing the oxygen consumption rate (OCR) levels in SH-SY5Y and BE(2)-C cells stably transfected with mock, *ecircCUX1*, *ecircCUX1* Mut, *p113*, sh-Scb, or sh-ecircCUX1, and those treated with BSA or oleic acid (OLE, 200 μmol·L^− 1^, *n* = 4). **h** Relative NAD^+^/NADH ratio and ATP levels in SH-SY5Y and BE(2)-C cells stably transfected with mock, *ecircCUX1*, *ecircCUX1* Mut, *p113*, sh-Scb, or sh-ecircCUX1, and those treated with BSA or OLE (200 μmol·L^− 1^, *n* = 5). ANOVA compared the difference in **f**-**h**. *P < 0.05 vs. mock, sh-Scb, or sh-Scb + BSA. Data are shown as mean ± s.e.m. (error bars) and representative of three independent experiments in **a**-**d**, **g** and **h**

Then, we observed the expression profiles of p113 in tumor tissues and cells. Higher p113 levels were noted in NB specimens and cell lines, than those of normal dorsal root ganglia (Fig. 2a). Endogenous p113 expression was noted in nuclei of tumor cells (Additional file 1: Fig. S2a), which was detected in 28/42 (66.7%) NB cases, with higher expression in those with elder age (*P* = 0.001), poor differentiation (*P* = 0.001), higher mitosis karyorrhexis index (*P* = 0.002), or advanced international neuroblastoma staging system (INSS) stages (*P* = 0.003, Additional file 1: Table S5). In these patients, high p113 expression was associated with poor survival probability (*P* < 1.0 × 10^− 4^, Additional file 1: Fig. S2b). Elevated p113 expression was also detected in a variety of cancer tissues (Additional file 1: Fig. S2c). Using SH-SY5Y, SK-N-SH, BE(2)-C, and IMR32 cells (with low or high p113 levels) as models, stable transfection of wild-type *ecircCUX1* or two independent shRNAs targeting junction site of *ecircCUX1* (sh-ecircCUX1) resulted in over-expression or silencing of *p113*, but not of *p200* or *p110* (Fig. 2b and Additional file 1: Fig. S2d), mainly localizing at nuclei (Fig. 2c, d). No alteration of p113 expression was observed in NB cells stably transfected with ORF mutant form of *ecircCUX1* (Fig. 2b).

Since previous studies implicate the potential impacts of SD on lipid metabolic reprogramming [26], lipid metabolite profiling assay was further undertaken, which revealed the increase of long-chain fatty acid production in SH-SY5Y cells stably transfected with *ecircCUX1* (Fig. 2e). Of note, over-expression of *ecircCUX1* or *p113*, but not of *ecircCUX1* with ORF mutation (*ecircCUX1* Mut), increased the levels of palmitic acid (C16:0), palmitoleic acid (C16:1n7), oleic acid (C18:1n9c), stearic acid (C18:0), and arachidonic acid (C20:4n6), while knockdown of *ecircCUX1* exerted the opposite effects (Fig. 2f). To explore the potential roles of *ecircCUX1* in FAO, NB cells were treated with oleic acid under fatty acid-restricted conditions. There was elevated OCR, mitochondrial membrane potential, complex I activity, NAD^+^/NADH ratio, and ATP production in SH-SY5Y and BE(2)-C cells stably transfected with *ecircCUX1* or *p113*, but not with *ecircCUX1* Mut (Fig. 2g, h and Additional file 1: Fig. S2e, f). Meanwhile, silencing of *ecircCUX1* abolished the increase of OCR, mitochondrial membrane potential, complex I activity, NAD^+^/NADH ratio, and ATP production induced by exogenous oleic acid (Fig. 2g, h and Additional file 1: Fig. S2e, f). However, ectopic expression of *ecircCUX1* or *p113* did not affect mitochondrial mass in NB cells, with intact two-layer membrane and inner structures (Additional file 1: Fig. S3a-c). These results suggested that p113 was upregulated and facilitated fatty acid oxidation and mitochondrial activity in NB.

### *ecircCUX1* promotes growth and aggressiveness of NB cells via encoding p113

We further explored the biological impacts of *ecircCUX1* on growth and aggressiveness of NB. Stable over-expression of *ecircCUX1* or *p113*, but not of *ecircCUX1* Mut, facilitated the growth and invasion capability of SH-SY5Y and SK-N-SH cells (Additional file 1: Fig. S4a-d). Meanwhile, the growth and invasion of BE(2)-C and IMR32 cells were significantly reduced by knockdown of *ecircCUX1* (Additional file 1: Fig. S4a-d). Consistently, stable transfection of *ecircCUX1* or sh-ecircCUX1 #2 into NB cells resulted in a significant increase or decrease of growth, tumor weight, long-chain fatty acid levels, complex I activity, NAD^+^/NADH ratio, ATP production, Ki-67 proliferation index, and CD31-positive microvessels of subcutaneous xenografts in athymic nude mice (Additional file 1: Fig. S4e, S5a, S5b). In experimental metastasis assay, athymic nude mice treated with tail vein injection of SH-SY5Y or BE(2)-C cells stably transfected with *ecircCUX1* or sh-ecircCUX1 #2 displayed more or fewer lung metastatic colonies, with lower or greater survival probability, respectively (Additional file 1: Fig. S5c, d). These results suggested that *ecircCUX1* promoted the growth and aggressiveness of NB cells via encoding p113*.*

### p113 physically interacts with ZRF1 and BRD4 in NB cells

**Fig. 3:**
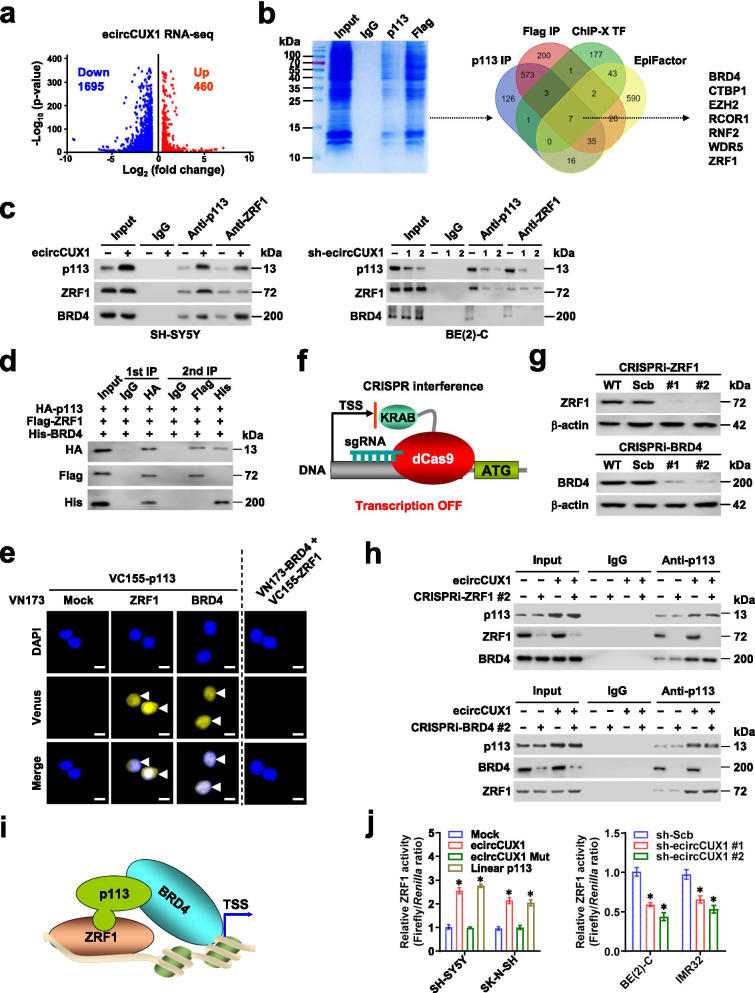
p113 physically interacts with ZRF1 and BRD4 in NB cells. **a** Volcano plots showing differentially expressed genes (fold change> 1.5, *P* < 0.05) in SH-SY5Y cells stably transfected with empty vector (mock) or *ecircCUX1*. **b** Coomassie blue staining (left panel) and Venn diagram (right panel) revealing identification of p113-interacting proteins pulled down by p113 or Flag-tag antibody in SH-SY5Y cells stably transfected with 3Flag-tagged *p113*, and those overlapped with transcription factors (TF) or epigenetic factors derived from ChIP-X and EpiFactors databases. **c** Co-IP and western blot assays indicating the interaction among p113, ZRF1, and BRD4 in SH-SY5Y and BE(2)-C cells stably transfected with mock, *ecircCUX1*, scramble shRNA (sh-Scb), or sh-ecircCUX1. **d** Secondary co-IP assays showing protein interaction among p113, ZRF1, and BRD4 in SH-SY5Y cells stably transfected with HA-tagged *p113*, Flag-tagged *ZRF1*, and His-tagged *BRD4*. **e** BiFC assay revealing the interaction of p113 with ZRF1 or BRD4 (arrowheads) in SH-SY5Y cells stably transfected with indicated constructs, with nuclei stained by DAPI. Scale bars: 10 μm. **f** and **g** Western blot assay (g) validating the knockdown of *ZRF1* or *BRD4* in SH-SY5Y cells stably transfected with scramble (Scb) or specific sgRNA for CRISPR interference (CRISPRi, f). Wild type (WT) cells were taken as negative controls. **h** Co-IP and western blot assays indicating the interaction of p113 with ZRF1 or BRD4 in SH-SY5Y cells stably transfected with mock or *ecircCUX1*, and those co-transfected with CRISPRi sgRNA specific against *ZRF1* or *BRD4*. **i** Schematic illustration of protein interaction among p113, ZRF1, and BRD4. **j** Dual-luciferase assay showing the activity of ZRF1 in NB cells stably transfected with mock, *ecircCUX1*, *ecircCUX1* Mut, *p113*, sh-Scb, or sh-ecircCUX1 (*n* = 5). Fisher’s exact test for overlapping analysis in **b**. ANOVA compared the difference in **j**. *P < 0.05 vs. mock or sh-Scb. Data are shown as mean ± s.e.m. (error bars) and representative of three independent experiments in **c**-**e**, **g**, **h** and **j**

To characterize downstream targets of p113, RNA-seq assay was performed, which indicated 460 upregulated and 1695 downregulated genes in SH-SY5Y cells upon *ecircCUX1* over-expression (Fig. 3a). Co-IP and subsequent mass spectrometry assays revealed 761 and 847 protein pulled down by p113 or Flag antibody in SH-SY5Y cells stably transfected with 3Flag-tagged *p113* (Additional file 1: Table S6 and Table S7), and overlapping analysis with transcription factors and epigenetic factors derived from ChIP-X program [27] and EpiFactors database [28] identified seven potential p113-interacting proteins (Fig. 3b). Among them, ZRF1 was the only transcription factor, while BRD4 might serve as a transcriptional co-factor. Endogenous interaction of p113 with ZRF1 and BRD4 was observed in NB cells, which was respectively increased or decreased by ectopic expression or knockdown of *ecircCUX1*, but not affected by transfection of *ecircCUX1* with ORF mutation (Fig. 3c and Additional file 1: Fig. S6a). Two-step immunoprecipitation assay revealed respective binding of p113 to ZRF1 or BRD4 (Fig. 3d). Especially, SANT domain (450-621 aa) of ZRF1 as well as bromodomain 2 (BD2) domain (250-470 aa) of BRD4 were essential for their interaction with p113 (Additional file 1: Fig. S6b-d). In BiFC assay [29], p113 physically interacted with ZRF1 and BRD4, whereas no interaction was noted between ZRF1 and BRD4 (Fig. 3e). Then, CRISPR interference (CRISPRi) approach [30] was applied for knockdown of *ZRF1* or *BRD4* (Fig. 3f, g). Ectopic expression of *ecircCUX1* increased the interaction of p113 with ZRF1 or BRD4, which was individually abolished by silencing of *ZRF1* or *BRD4*, respectively (Fig. 3h), indicating the mediating roles of p113 in forming trimer complex with ZRF1 and BRD4 (Fig. 3i). Notably, ectopic expression or knockdown of *ecircCUX1* facilitated and attenuated the transactivation of ZRF1 in NB cells, respectively (Fig. 3j). Meanwhile, ectopic expression of *ecircCUX1* with ORF mutation did not affect transcriptional activity of ZRF1 (Fig. 3j). These results indicated that p113 physically interacted with ZRF1 and BRD4 as a trimer complex in NB cells.

### p113/ZRF1/BRD4 complex promotes lipid metabolic reprogramming and mitochondrial complex I activity in NB cells

**Fig. 4:**
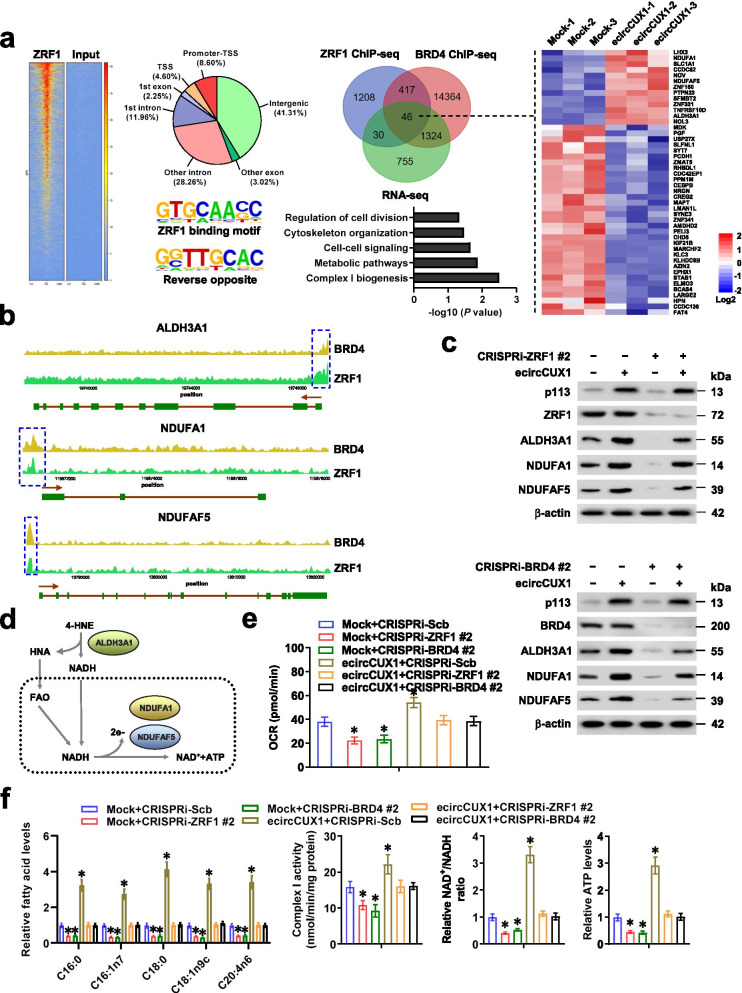
p113/ZRF1/BRD4 complex promotes lipid metabolic reprogramming and mitochondrial complex I activity in NB cells. **a** Heatmap, distribution, and binding motif of ChIP-Seq (left panel) assay revealing genomic enrichment of ZRF1 in SH-SY5Y cells, while Venn diagram, heatmap, and GO pathway (right panel) showing identification of p113/ZRF1/BRD4 target genes by overlapping analysis of RNA-seq results upon *p113* over-expression and ChIP-seq peaks of ZRF1 or BRD4. **b** ChIP-seq assay showing the binding peak of BRD4 or ZRF1 on promoter regions of *ALDH3A1*, *NDUFA1*, or *NDUFAF5* in SH-SY5Y cells. **c** Western blot assay indicating the expression of ALDH3A1, NDUFA1, or NDUFAF5 in SH-SY5Y cells stably transfected with empty vector (mock) or *ecircCUX1*, and those co-transfected with sgRNA specific against *ZRF1* or *BRD4* for CRISPRi. **d** Schematic illustration showing the involvement of ALDH3A1, NDUFA1, or NDUFAF5 in lipid metabolic reprogramming and mitochondrial respiratory activity. **e** Relative OCR levels in SH-SY5Y cells stably transfected with mock or *ecircCUX1*, and those co-transfected with sgRNA specific against *ZRF1* or *BRD4* for CRISPRi (*n* = 5). **f** Relative fatty acid levels, complex I activity, NAD^+^/NADH ratio, and ATP levels in SH-SY5Y cells stably transfected with mock or *ecircCUX1*, and those co-transfected with sgRNA specific against *ZRF1* or *BRD4* for CRISPRi (*n* = 5). ANOVA compared the difference in **e** and **f**. *P < 0.05 vs. mock+CRISPRi-Scb. Data are shown as mean ± s.e.m. (error bars) and representative of three independent experiments in **c**, **e** and **f**

To further investigate target genes of p113/ZRF1/BRD4 complex, ChIP-seq assay was performed using ZRF1- or BRD4-specific antibody, while results were overlappingly analyzed with *ecircCUX*-regulated genes derived from RNA-seq. Comprehensive analysis revealed 46 target genes regulated by transcriptional trimer complex p113/ZRF1/BRD4, especially those involved in metabolic pathway or complex I biogenesis, including *ALDH3A1*, *NDUFA1*, and *NDUFAF5* (Fig. 4a, b). Knockdown of *ZRF1* abolished the increased enrichment of p113/ZRF1/BRD4 complex on promoters of these target genes induced by over-expression of *ecircCUX1*, while silencing of *BRD4* did not affect the binding of p113 or ZRF1 to target gene promoters (Additional file 1: Fig. S7a-c). Ectopic expression of *ecircCUX* resulted in increase of promoter activity, transcript and protein levels of *ALDH3A1*, *NDUFA1*, and *NDUFAF5* in SH-SY5Y and SK-N-SH cells, which were eliminated after knockdown of *ZRF1* or *BRD4*, respectively (Additional file 1: Fig. S7a-c and Fig. 4c). Consistent with the roles of *ALDH3A1* in converting lipid peroxidation product (4-HNE) into fatty acids [31] (Fig. 4d), immunostaining assay indicated upregulation of ALDH3A1 and downregulation of 4-HNE in NB cells stably over-expressing *ecircCUX*, which was rescued by silencing of *ZRF1* or *BRD4* (Additional file 1: Fig. S8a). Notably, silencing of *ZRF1* or *BRD4* abolished the increase of long-chain fatty acid production, OCR levels, mitochondrial membrane potential, complex I activity, NAD^+^/NADH ratio, and ATP production in SH-SY5Y cells stably over-expressing *ecircCUX1* (Fig. 4e, f and Additional file 1: Fig. S8b). Moreover, ectopic expression of *ZRF1* increased the expression of *ALDH3A1*, *NDUFA1*, and *NDUFAF5*, and led to increase in OCR levels, mitochondrial membrane potential, complex I activity, NAD^+^/NADH ratio, ATP production, growth, and invasion of BE(2)-C cells, while knockdown of *ALDH3A1*, *NDUFA1*, or *NDUFAF5* abolished these effects (Additional file 1: Fig. S9a-f). Taken together, these results suggested that p113/ZRF1/BRD4 complex promoted lipid metabolic reprogramming and mitochondrial complex I activity in NB cells.

### p113 facilitates tumorigenesis and aggressiveness of NB cells via interacting with ZRF1

**Fig. 5:**
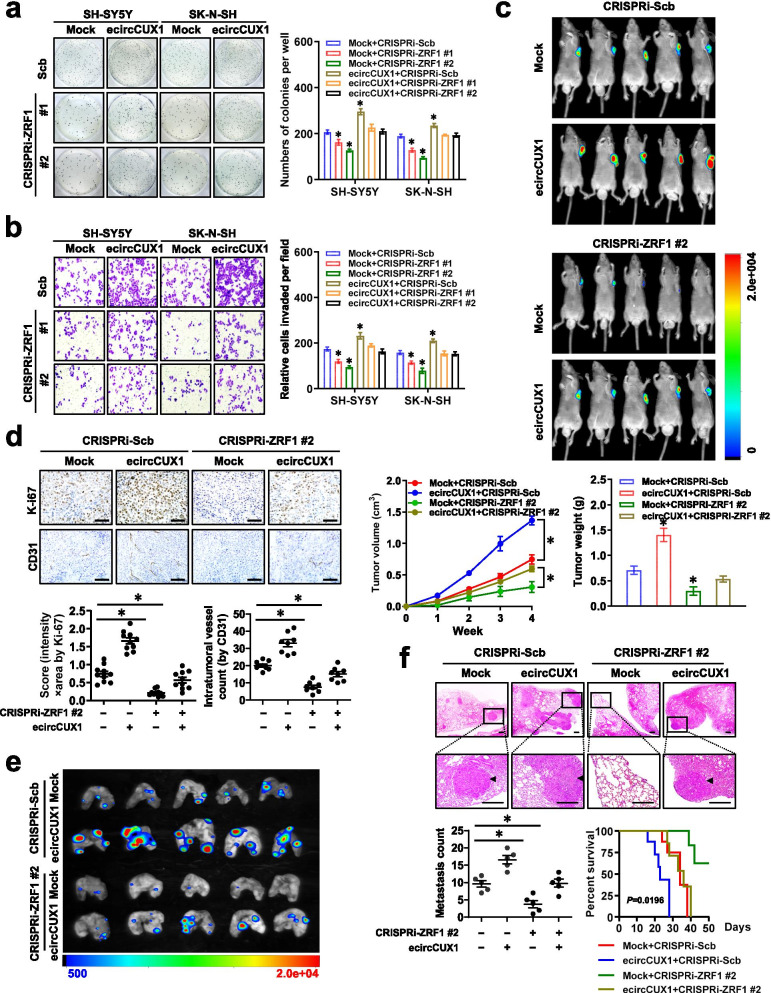
p113 facilitates tumorigenesis and aggressiveness of NB cells via interacting with ZRF1. **a** and **b** Representative images (left panel) and quantification (right panel) of soft agar (a) and matrigel invasion (b) assays indicating the growth and invasion of NB cells stably transfected with empty vector (mock) or *ecircCUX1*, and those co-transfected with sgRNA specific against *ZRF1* for CRISPRi. **c** Representative images (upper panel), in vivo growth curve, and weight at the end points (lower panel) of xenografts in nude mice formed by subcutaneous injection of SH-SY5Y cells stably transfected with mock or *ecircCUX1*, and those co-transfected with sgRNA specific against *ZRF1* for CRISPRi (*n* = 5 for each group). **d** Representative images (upper panel) and quantification (lower panel) of immunohistochemical staining showing expression of Ki-67 and CD31 within xenografts (*n* = 5 for each group). Scale bars: 50 μm. **e** and **f** Representative in vivo images (e), hematoxylin & eosin staining (f, upper panel), lung metastatic counts (f, lower panel), and Kaplan–Meier curves (f, lower panel) of nude mice treated with tail vein injection of SH-SY5Y cells stably transfected with mock or *ecircCUX1*, and those co-transfected with sgRNA specific against *ZRF1* for CRISPRi (*n* = 5 for each group). Scale bars: 100 μm. ANOVA compared the difference in **a**-**d** and **f**. Log-rank test for survival comparison in **f**. *P < 0.05 vs. mock+CRISPRi-Scb. Data are shown as mean ± s.e.m. (error bars) and representative of three independent experiments in **a** and **b**

We further explored the cooperative roles of p113 and ZRF1 in NB progression. Knockdown of *ZRF1* by CRISPRi significantly prevented the increase of anchorage-independent growth and invasion of NB cells induced by stable *ecircCUX1* over-expression (Fig. 5a, b). There were increased growth, volume, weight, Ki-67 proliferation index, and CD31-positive intratmoral microvessels of subcutaneous xenografts in athymic nude mice formed by injection of SH-SY5Y cells stably transfected with *ecircCUX1*, and these effects were eliminated after knockdown of *ZRF1* (Fig. 5c, d). Athymic nude mice treated with tail vein injection of SH-SY5Y cells stably transfected with *ecircCUX1* presented more lung metastatic colonies and lower survival probability, which was abolished by silencing of *ZRF1* (Fig. 5e, f). These results suggested that p113 facilitated the tumorigenesis and aggressiveness of NB cells via interacting with ZRF1.

### Therapeutic blocking p113-ZRF1 interaction inhibits NB progression

**Fig. 6:**
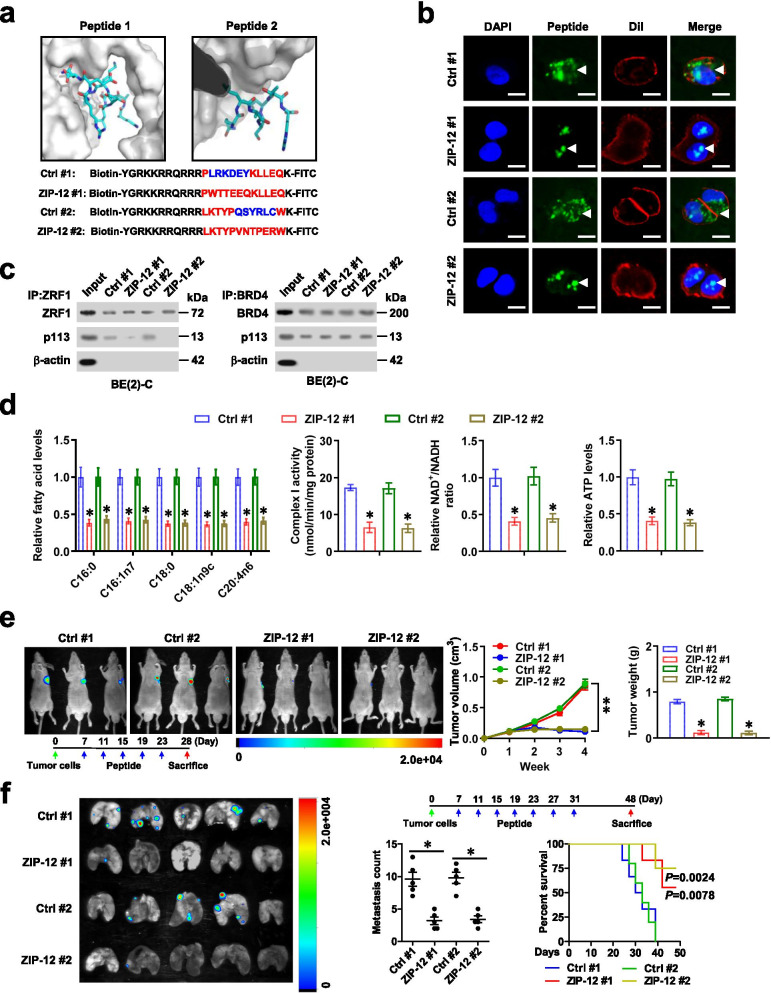
Therapeutic blocking p113-ZRF1 interaction inhibits NB progression. **a** 3D structure and sequences of inhibitory peptides (ZIP-12) for blocking interaction between p113 and ZRF1, and those of mutant control (Ctrl) peptides. **b** Confocal images showing the distribution of synthesized FITC-labeled Ctrl or ZIP-12 peptides (20 μmol·L^− 1^, arrowheads) within cultured BE(2)-C cells, with nuclei and cellular membranes staining with DAPI or Dil. Scale bars: 10 μm. **c** Co-IP and western blot assays indicating the interaction of p113 with ZRF1 or BRD4 in BE(2)-C cells treated with Ctrl or ZIP-12 peptides (20 μmol·L^− 1^) for 24 h. **d** Relative fatty acid levels, complex I activity, NAD^+^/NADH ratio, and ATP levels in BE(2)-C cells treated with Ctrl or ZIP-12 peptides (20 μmol·L^− 1^) for 24 h. **e** In vivo images (left upper panel), growth curve (right panel), and weight at the end points (right panel) of xenografts formed by subcutaneous injection of BE(2)-C cells in nude mice (*n* = 5 per group) that were treated with intravenous injection of Ctrl or ZIP-12 peptides (5 mg·kg^− 1^) as indicated (left lower panel). **f** In vivo imaging (left panel), lung metastatic colonization (right lower panel), and Kaplan–Meier curves (right lower panel) of nude mice (*n* = 5 for each group) treated with tail vein injection of BE(2)-C cells, Ctrl or ZIP-12 peptides (5 mg·kg^− 1^) as indicated (right upper panel). Student’s *t* test or ANOVA compared the difference in **d**-**f**. Log-rank test for survival comparison in **f**. *P < 0.05, **P < 0.01 vs. Ctrl. Data are shown as mean ± s.e.m. (error bars) and representative of three independent experiments in **b**-**d**

Based on the importance of SANT domain of ZRF1 in interacting with p113, we designed cell-penetrating peptides using Peptiderive server [32], named as ZRF1 inhibitory peptide with 12 amino acids in length (ZIP-12) (Fig. 6a). Incubation of NB cells with ZIP-12, but not with mutant control peptide, led to obvious aggregation within nucleus (Fig. 6b). ZIP-12 was able to bind to endogenous p113 protein (Additional file 1: Fig. S10a) and abolish the interaction between p113 and ZRF1 in NB cells, without impact on that of p113 and BRD4 (Fig. 6c). In addition, treatment with ZIP-12, but not of mutant control peptide, inhibited the ZRF1 enrichment, promoter activity, and expression of *ALDH3A1*, *NDUFA1*, and *NDUFAF5* in NB cells (Additional file 1: Fig. S10b-e). As a result, there was reduced long-chain fatty acid levels, mitochondrial membrane potential, complex I activity, NAD^+^/NADH ratio, ATP production in ZIP-12-treated BE(2)-C cells (Additional file 1: Fig. S10f and Fig. 6d). Administration of ZIP-12 suppressed the viability of BE(2)-C and IMR32 cells in a dose- and time-dependent manner, without impact on that of non-transformed normal MCF 10A cells (Additional file 1: Fig. S11a, b). Moreover, ZIP-12 repressed anchorage-independent growth and invasion of BE(2)-C and IMR32 cells (Additional file 1: Fig. S11c). Intravenous administration of ZIP-12 suppressed the fluorescence signals, volume, weight, Ki-67 proliferation index, and CD31-positive microvessels of subcutaneous xenograft tumors formed by BE(2)-C cells in nude mice (Fig. 6e and Additional file 1: Fig. S11d). Moreover, intravenous administration of ZIP-12 led to less lung metastatic colonies and prolonged the survival time of athymic nude mice treated with tail vein injection of BE(2)-C cells (Fig. 6f and Additional file 1: Fig. S11e). Collectively, these results demonstrated that blocking p113-ZRF1 interaction suppressed NB progression.

### *CUX1*, *ZRF1*, *BRD4* and target genes are associated with poor outcome of tumor patients

**Fig. 7:**
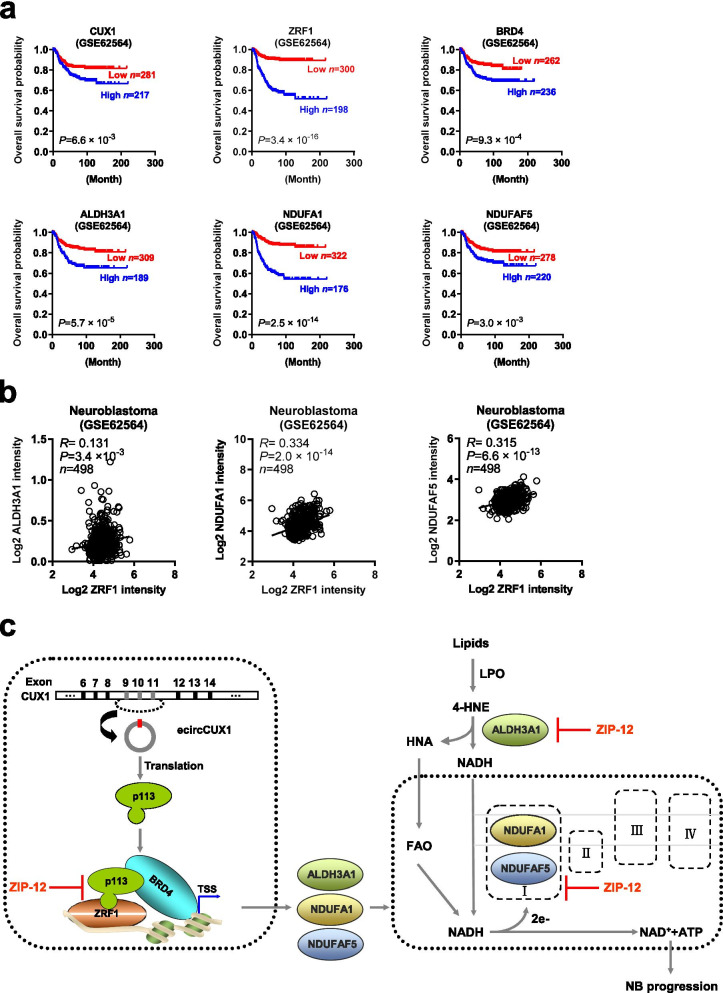
*CUX1*, *ZRF1*, *BRD4* and target genes are associated with poor outcome of NB patients. **a** Kaplan–Meier curves indicating overall survival of 498 well-defined NB cases (GSE62564) with high or low expression of *CUX1* (cutoff value = 32.233), *ZRF1* (cutoff value = 21.749), *BRD4* (cutoff value = 652.58), *ALDH3A1* (cutoff value = 1.181), *NDUFA1* (cutoff value = 22.511), or *NDUFAF5* (cutoff value = 7.964). **b** The positive expression correlation between *ZRF1* and *ALDH3A1*, *NDUFA1*, or *NDUFAF5* in 498 well-defined NB cases (GSE62564). **c** The mechanisms underlying p113-faciliated NB progression: as a novel protein encoded by *ecircCUX1*, p113 cooperates with ZRF1 and BRD4 to activate the transcription of *ALDH3A1*, *NDUFA1*, or *NDUFAF5*, resulting in promoted conversion of fatty aldehydes into fatty acids, fatty acid β-oxidation, mitochondrial complex I activity, growth, and aggressiveness of NB cells. Meanwhile, inhibitory peptides (ZIP-12) blocking p113-ZRF1 interaction suppresses tumor progression. Log-rank test for survival comparison in **a**. Pearson’s correlation coefficient for **b**

In 498 well-defined NB cases (GSE62564), Kaplan–Meier survival plots revealed that higher expression of p113 host gene *CUX1* (*P* = 6.6 × 10^− 3^), *ZRF1* (*P* = 3.4 × 10^− 16^), *BRD4* (*P* = 9.3 × 10^− 4^), *ALDH3A1* (*P* = 5.7 × 10^− 5^), *NDUFA1* (*P* = 2.5 × 10^− 14^), or *NDUFAF5* (*P* = 3.0 × 10^− 3^) was associated with lower overall survival probability of patients (Fig. 7a). High expression of *ZRF1* or *BRD4* was also associated with poor survival of patients with breast cancer, colon cancer, Ewing sarcoma, glioma, melanoma, myeloma, or renal cancer (Additional file 1: Fig. S12). There was a positive expression correlation between *ZRF1* and *ALDH3A1* (*R* = 0.131, *P* = 3.4 × 10^− 3^), *NDUFA1* (*R* = 0.334, *P* = 2.0 × 10^− 14^), or *NDUFAF5* (*R* = 0.315, *P* = 6.6 × 10^− 13^) in 498 NB specimens (Fig. 7b). These results indicated that expression of *CUX1*, *ZRF1*, *BRD4*, or target genes was associated with poor outcome of tumors.

## Discussion

As a homeodomain transcription factor, CUX1 regulates cell cycle progression, proliferation, migration, and invasiveness of numerous tumor cell lines [33]. The major isoforms of CUX1 include full-length p200, p110 proteolytically processed from p200, and CASP [34]. Notably, p200 binds rapidly but only transiently to DNA, while p110 isoform stably interacts with DNA to regulate gene transcription [35]. Meanwhile, CASP is a Golgi membrane protein related to giantin [36]. In our previous study, we have demonstrated oncogenic roles of *CUX1* and its generated circRNA in aerobic glycolysis and tumor progression [19]. In this study, through high-throughput quantitative proteomics, we discovered that a new CUX1 isoform (p113), but not p200, p110 or CASP, was induced by serum loss stress in NB cells. As a protein encoded by *ecircCUX1*, p113 was upregulated in NB tissues and cell lines, and associated with poor survival of NB patients. Gain- and loss-of-function studies indicated that p113 promoted the lipid metabolism, mitochondrial complex I activity, growth, and invasiveness of NB cells (Fig. 7c), suggesting its oncogenic roles in NB progression.

In tumor cells, there was spontaneous fatty acid peroxidation mediated by reactive oxygen species, resulting in production of fatty aldehydes [37]. As an unique process, ALDH3A1 catalyzes conversion of fatty aldehydes to fatty acids with NADH production as an extra source of electrons, resulting in mitochondrial FAO and ATP synthesis [38]. It is well known that mitochondrial ETC is necessary for tumor growth [39]. As the largest component of mitochondrial respiratory chain, complex I is the first entry point of electrons into oxidative phosphorylation, and donates electrons to ubiquinone, resulting in generation of ubiquinol and NAD^+^ to maintain cellular NAD^+^/NADH balance [40]. As a component of complex I, NDUFA1 is essential for respiratory activity [41] and tumor growth [42]. NDUFAF5 is involved in building up complex I and electrochemical potential required for ATP production [43]. In this study, we demonstrated that p113 induced transcriptional upregulation of *ALDH3A1*, *NDUFA1*, and *NDUFAF5*, leading to increased conversion of fatty aldehydes into fatty acids, fatty acid β-oxidation, and mitochondrial complex I activity for ATP production, providing a novel mechanism regulating lipid metabolic reprogramming and mitochondrial activity in NB. In addition, our evidence indicated that p113 directly interacted with transcription factor ZRF1. Since knockdown of *ZRF1* abolished the p113-facilitated aggressive behavior of NB cells, our findings indicate that tumor promoting functions of *p113* are mediated, at least in part, through interacting with ZRF1.

ZRF1 is a transcription factor recognizing target genes via DNAJ or SANT domain [44], and is involved in various biological processes such as embryonic development, cell differentiation, neural progenitor cell maintenance, apoptosis, and cellular proliferation [45]. In addition, ZRF1 attenuates the recruitment of polycomb repressive complex 1 (PRC1) on gene promoters by binding to mono-ubiquitinated histone H2A, resulting in de-repression of PRC1 downstream genes [46]. Elevated *ZRF1* expression is documented in many tumors, and is associated with poor survival of tumor patients [45, 47], suggesting its oncogenic roles in tumor progression. As a transcriptional co-activator, BRD4 is one member of BD domain and ET domain family, and preferentially interacts with acetylated lysine of histones to recruit distinct transcriptional regulators, leading to initiation and elongation of gene transcription [48]. BRD4 plays a pivotal role in embryogenesis and cancer development [49], while its inhibitor is able to eliminate transcriptional activation of oncogenes and suppress tumor progression [50]. In this study, we found that p113/ZRF1/BRD4 transcriptional trimer was essential for upregulation of genes involved in lipid metabolic reprogramming and mitochondrial activity. Meanwhile, silencing of *ZRF1* abolished the enrichment of this complex on target gene promoters. Importantly, an inhibitory peptide blocking p113-ZRF1 interaction was efficient in suppressing lipid metabolic reprogramming, mitochondrial activity, tumorigenesis, and aggressiveness of NB cells.

## Conclusions

In summary, we demonstrate that as a protein encoded by circRNA derived from *CUX1*, p113 is highly expressed in NB tissues and cells, and facilitates growth, invasion, and metastasis of NB cells. Mechanistically, p113 interacts with ZRF1 and BRD4 to form a transcriptional regulatory complex, resulting in transcriptional activation of *ALDH3A1*, *NDUFA1*, and *NDUFAF5*, thus enhancing lipid metabolic reprogramming and mitochondrial activity of NB cells. Importantly, in clinical NB cases, high p113 expression is associated with unfavorable survival probability. The inhibitory peptides blocking p113-ZRF1 interaction suppresses the tumorigenesis and aggressiveness of NB cells. We believe that this study extends our knowledge about the regulation of lipid metabolic reprogramming and mitochondrial activity by circRNA-coding protein, and reveals p113/ZRF1/BRD4 axis as a potential therapeutic target for NB progression.

## Supplementary Information

**Additional file 1: Fig. S1.** Coding ability of *ecircCUX1* in NB. **Fig. S2.** Expression profiles and roles of *ecircCUX1*-encoded p113. **Fig. S3.** Effects of *ecircCUX1* or *p113* on mitochondrial mass and structure in NB cells. **Fig. S4.** *ecircCUX1* promotes fatty acid oxidation and growth of NB via encoding p113. **Fig. S5.** *ecircCUX1* facilitates mitochondrial complex I activity and aggressiveness of NB in vivo. **Fig. S6.** Interaction of p113 with ZRF1 and BRD4 in NB cells. **Fig. S7.** *ecircCUX1* facilitates expression of *ALDH3A1*, *NDUFA1*, or *NDUFAF5* in NB cells. **Fig. S8.** *ecircCUX1* reduces peroxidated lipids and increases mitochondrial membrane potential via ZRF1 or BRD4 in NB cells. **Fig. S9.** *ZRF1* promotes mitochondrial complex I activity, growth, and aggressiveness of NB cells via target genes. **Fig. S10.** Effects of inhibitory peptides blocking p113-ZRF1 interaction. **Fig. S11.** ZIP-12 inhibits viabilities, growth, invasion, and metastasis of NB cells. **Fig. S12.** Kaplan-Meier curves of *ZRF1* and *BRD4* in multiple cancers. **Table S1.** Primer sets used for RT-PCR, qPCR and ChIP. **Table S2.** Oligonucleotide sets used for constructs. **Table S3.** Oligonucleotide sets used for short hairpin RNAs and CRISPR-Cas9/dCas9. **Table S4.** Mass spectrometry analysis of proteins altered by serum deprivation. **Table S5.** p113 expression in human NB tissues. **Table S6.** Mass spectrometry analysis of proteins pulled down by p113 antibody. **Table S7.** Mass spectrometry analysis of proteins pulled down by Flag antibody.

## Data Availability

The data supporting the conclusions of this article are presented within article and its additional files.

## Abbreviations

4-HNE: 4-Hydroxynonenal
ALDH3A1: Aldehyde dehydrogenase 3 family member A1
BiFC: Bimolecular fluorescence complementation
BRD4: Bromodomain protein 4
circRNA: Circular RNA
co-IP: Co-immunoprecipitation
CRISPRi: CRISPR interference
CUX1: CUT-like homeobox 1
ETC: Electron transport chain
FAO: Fatty acid β-oxidation
IRES: Internal ribosome entry site
NAD^+^: Nicotinamide adenine dinucleotide
NADH: Nicotinamide adenine dinucleotide
NB: Neuroblastoma
NDUFA1: NADH:ubiquinone oxidoreductase subunit A1
NDUFAF5: NADH:ubiquinone oxidoreductase complex assembly factor 5
OCR: Oxygen consumption rate
TCF3: Transcription factor 3
ZRF1: Zuotin-related factor 1.
