# Supplementary material for: p113 isoform encoded by CUX1 circular RNA drives tumor progression via facilitating ZRF1/BRD4 transactivation
Source: Mol Cancer. 2021 Sep 27;20:123. doi: 10.1186/s12943-021-01421-8 (PMC8474885; doi:10.1186/s12943-021-01421-8)
Supplement: Supplementary file 1 — Additional file 1: Fig. S1. Coding ability of ecircCUX1 in NB. Fig. S2. Expression profiles and roles of ecircCUX1-encoded p113. Fig. S3. Effects of ecircCUX1 or p113 on mitochondrial mass and structure in NB cells. Fig. S4. ecircCUX1 promotes fatty acid oxidation and growth of NB via encoding p113. Fig. S5. ecircCUX1 facilitates mitochondrial complex I activity and aggressiveness of NB in vivo. Fig. S6. Interaction of p113 with ZRF1 and BRD4 in NB cells. Fig. S7. ecircCUX1 facilitates expression of ALDH3A1, NDUFA1, or NDUFAF5 in NB cells. Fig. S8. ecircCUX1 reduces peroxidated lipids and increases mitochondrial membrane potential via ZRF1 or BRD4 in NB cells. Fig. S9. ZRF1 promotes mitochondrial complex I activity, growth, and aggressiveness of NB cells via target genes. Fig. S10. Effects of inhibitory peptides blocking p113-ZRF1 interaction. Fig. S11. ZIP-12 inhibits viabilities, growth, invasion, and metastasis of NB cells. Fig. S12. Kaplan-Meier curves of ZRF1 and BRD4 in multiple cancers. Table S1. Primer sets used for RT-PCR, qPCR and ChIP. Table S2. Oligonucleotide sets used for constructs. Table S3. Oligonucleotide sets used for short hairpin RNAs and CRISPR-Cas9/dCas9. Table S4. Mass spectrometry analysis of proteins altered by serum deprivation. Table S5. p113 expression in human NB tissues. Table S6. Mass spectrometry analysis of proteins pulled down by p113 antibody. Table S7. Mass spectrometry analysis of proteins pulled down by Flag antibody. [file 12943_2021_1421_MOESM1_ESM.pdf]

Supplementary Information

p113 isoform encoded by *CUX1* circular RNA drives tumor progression via facilitating ZRF1/BRD4 transactivation

Yang et al.

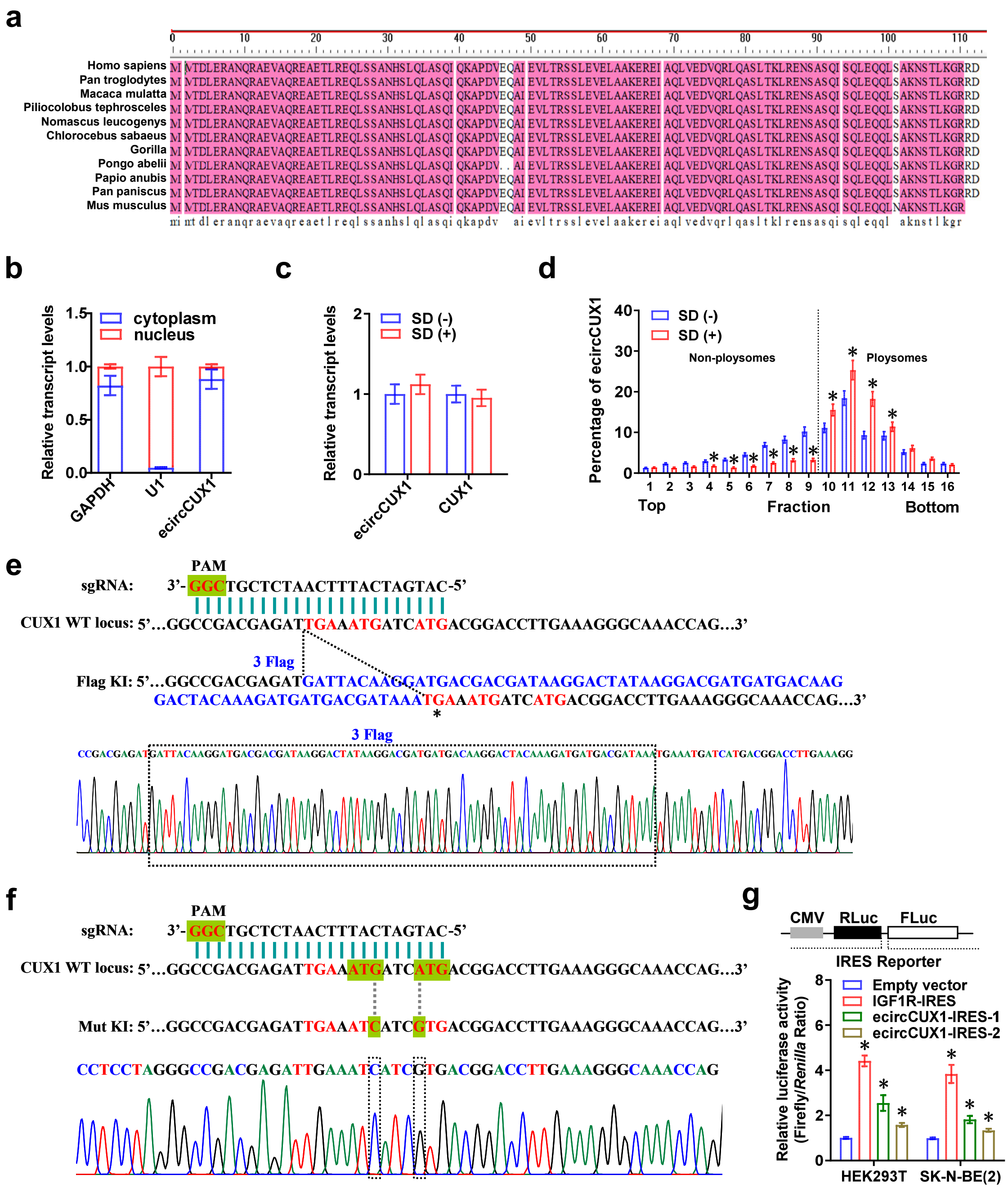

Supplementary Figure S1. Coding ability of *ecircCUX1* in NB. a Homologous analysis of protein encoded by open reading frame within *ecircCUX1*. **b** Real-time quantitative RT-PCR assay indicating the levels (normalized to β-actin) of *ecircCUX1*, *GAPDH*, and *U1* in the cytoplasmic and nuclear fractions of BE(2)-C cells (*n*=4). **c** Real-time qRT-PCR assay revealing the relative levels of *ecircCUX1* or *CUX1* (normalized to β-actin) in SH-SY5Y cells treated with serum deprivation (SD) for 24 hrs (*n*=5). **d** Sucrose gradient sedimentation assay showing the relative fraction of *ecircCUX1* binding to polysomes in SH-SY5Y cells treated with SD for 24 hrs (*n*=5). **e** and **f** sgRNA-targeting genomic region and Sanger sequencing validation for CRISPR-Cas9-mediated knockin of 3Flag-tagged (e) or mutant (f) *ecircCUX1*. **g** Dual-luciferase assay indicating the IRES reporter activity of *ecircCUX1* in HEK293T and SK-N-BE(2) cells, while that of *IGF1R* served as a positive control (*n*=4). Student's *t* test and ANOVA compared the difference in **c**, **d** and **g**. *$P<0.05$ vs. SD (-) or empty vector. Data are shown as mean ± s.e.m. (error bars) and representative of three independent experiments in **b**-**g**.

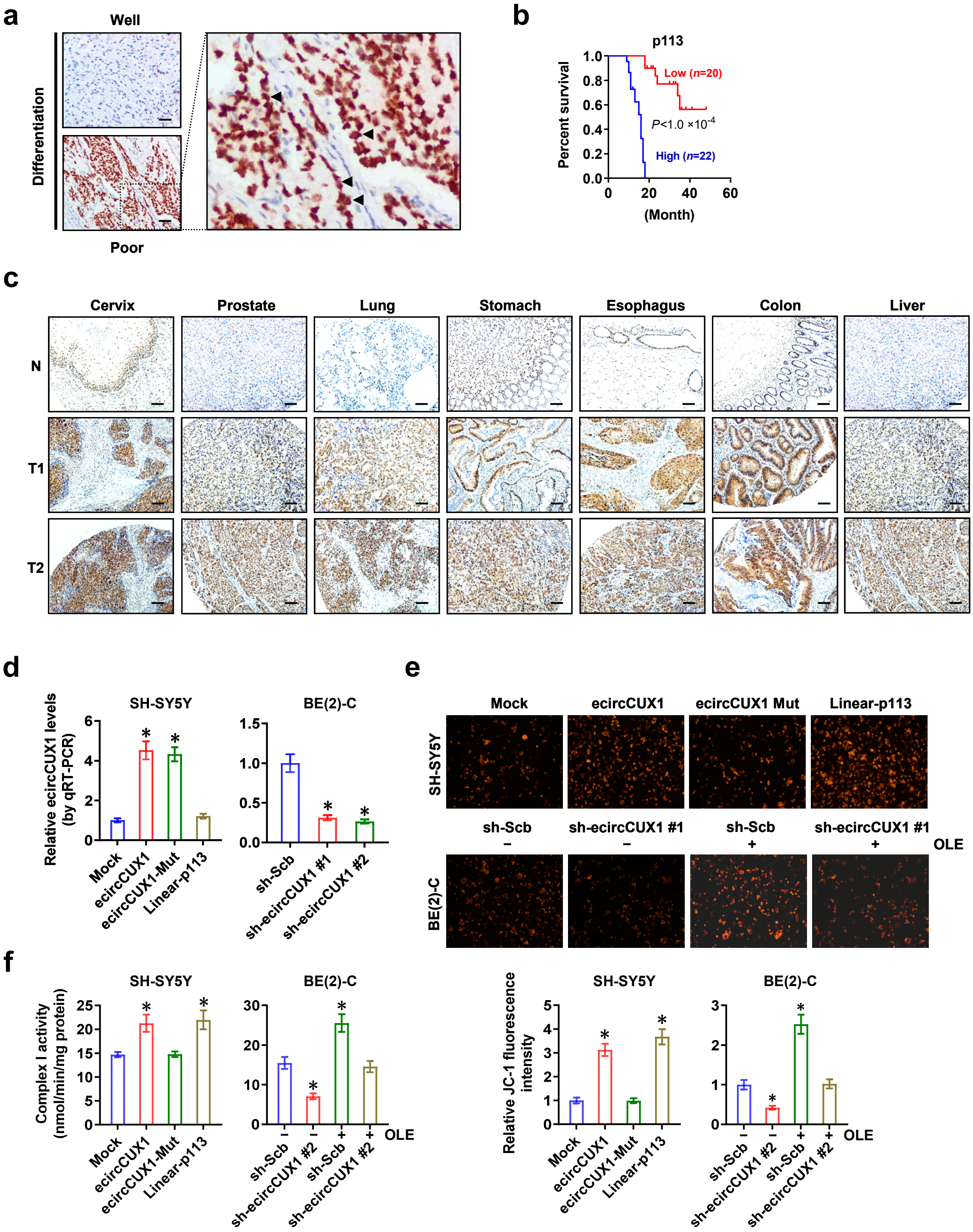

Supplementary Figure S2. Expression profiles and roles of *ecircCUX1*-encoded p113. a Immunohistochemical staining showing p113 expression in NB tissues (arrowheads). Scale bars: 100 μm. **b** Kaplan–Meier curves indicating overall survival of 42 well-defined NB cases with high or low expression of p113 (cutoff value=3.888). **c** Immunohistochemical staining showing p113 expression in tumoral (T) and normal (N) counterparts of common cancers (arrowheads). Scale bars: 100 μm. **d** Real-time qRT-PCR assay revealing the relative levels of *ecircCUX1* (normalized to β-actin) in SH-SY5Y cells stably transfected with empty vector (mock), *ecircCUX1*, *ecircCUX1* with ORF mutation (*ecircCUX1* Mut), or *p113* (n=5). **e** Representative images (left panel) and quantification (right panel) of JC-1 stained SH-SY5Y and BE(2)-C cells stably transfected with mock, *ecircCUX1*, *ecircCUX1* Mut, *p113*, scramble shRNA (sh-Scb), or sh-ecircCUX1, and those treated with BSA or oleic acid (OLE, 200 $\mu mol \cdot L^{-1}$, n=4). **f** Relative complex I activity in SH-SY5Y and BE(2)-C cells stably transfected with mock, *ecircCUX1*, *ecircCUX1* Mut, *p113*, sh-Scb, or sh-ecircCUX1 (n=5). ANOVA compared the difference in **d-f**. Log-rank test for survival comparison in **b**. *P< 0.05 vs. mock or sh-Scb. Data are shown as mean ± s.e.m. (error bars) and representative of three independent experiments in **d-f**.

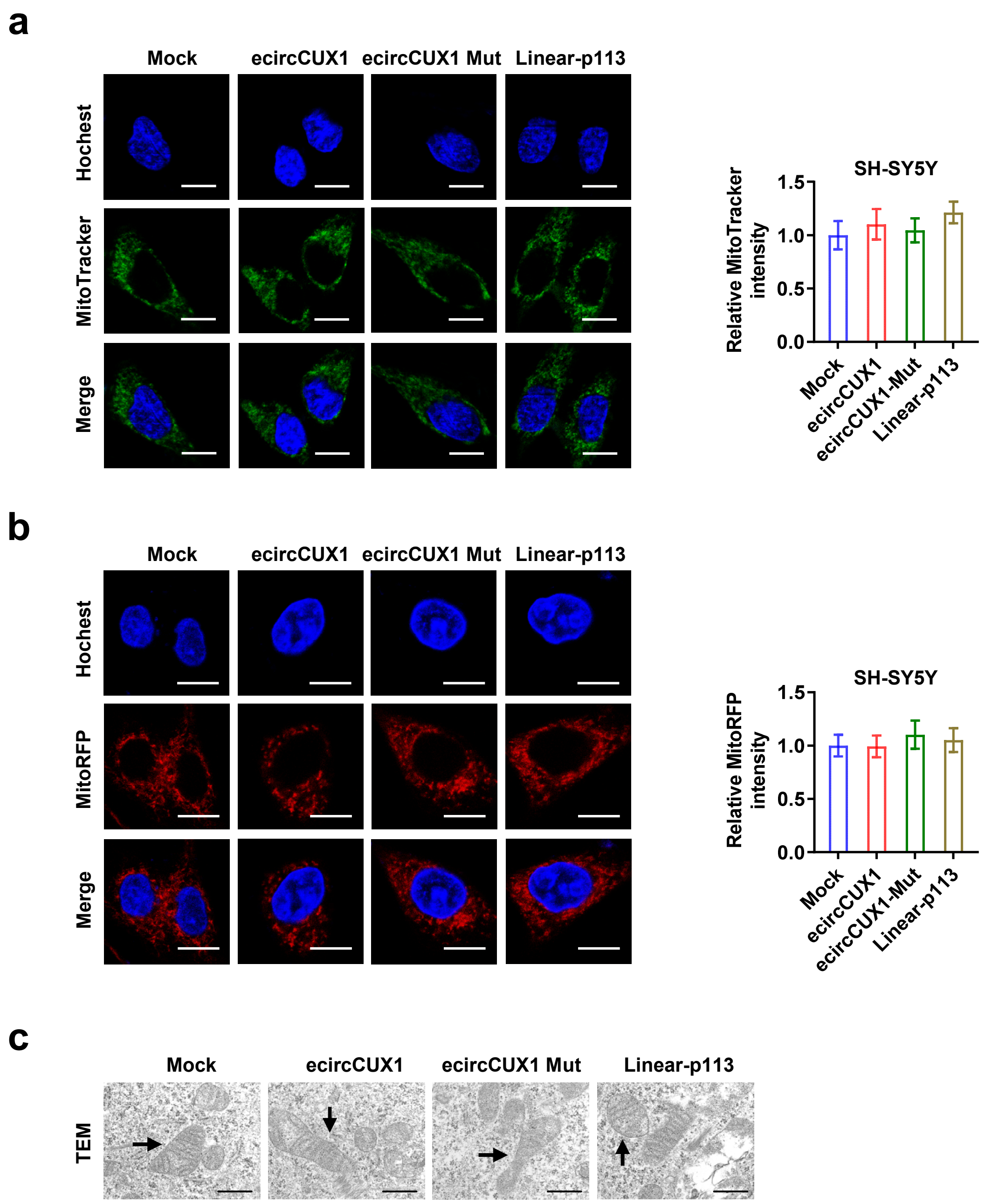

Supplementary Figure S3. Effects of *ecircCUX1* or *p113* on mitochondrial mass and structure in NB cells. a Representative images and quantification of MitoTracker Green staining (100 nmol·L^{-1}, at 37°C for 30 min) revealing mitochondrial mass in SH-SY5Y cells stably transfected with empty vector (mock), *ecircCUX1*, ecircCUX1 with ORF mutation (*ecircCUX1* Mut), or *p113*, with nuclei stained by Hochest 33342. Scale bar, 10 μm. **b** Representative images and quantification of MitoRFP reporter-indicated mitochondrial mass in SH-SY5Y cells stably transfected with mock, *ecircCUX1*, *ecircCUX1* Mut, or *p113*, with nuclei stained by Hochest 33342. Scale bar, 10 μm. **c** Transmission electron microscopy (TEM) showing mitochondrial structure in SH-SY5Y cells stably transfected with mock, *ecircCUX1*, *ecircCUX1* Mut, or *p113*. ANOVA compared the difference in **a** and **b**. Data are shown as mean ± s.e.m. (error bars) and representative of three independent experiments in **a** and **b**.

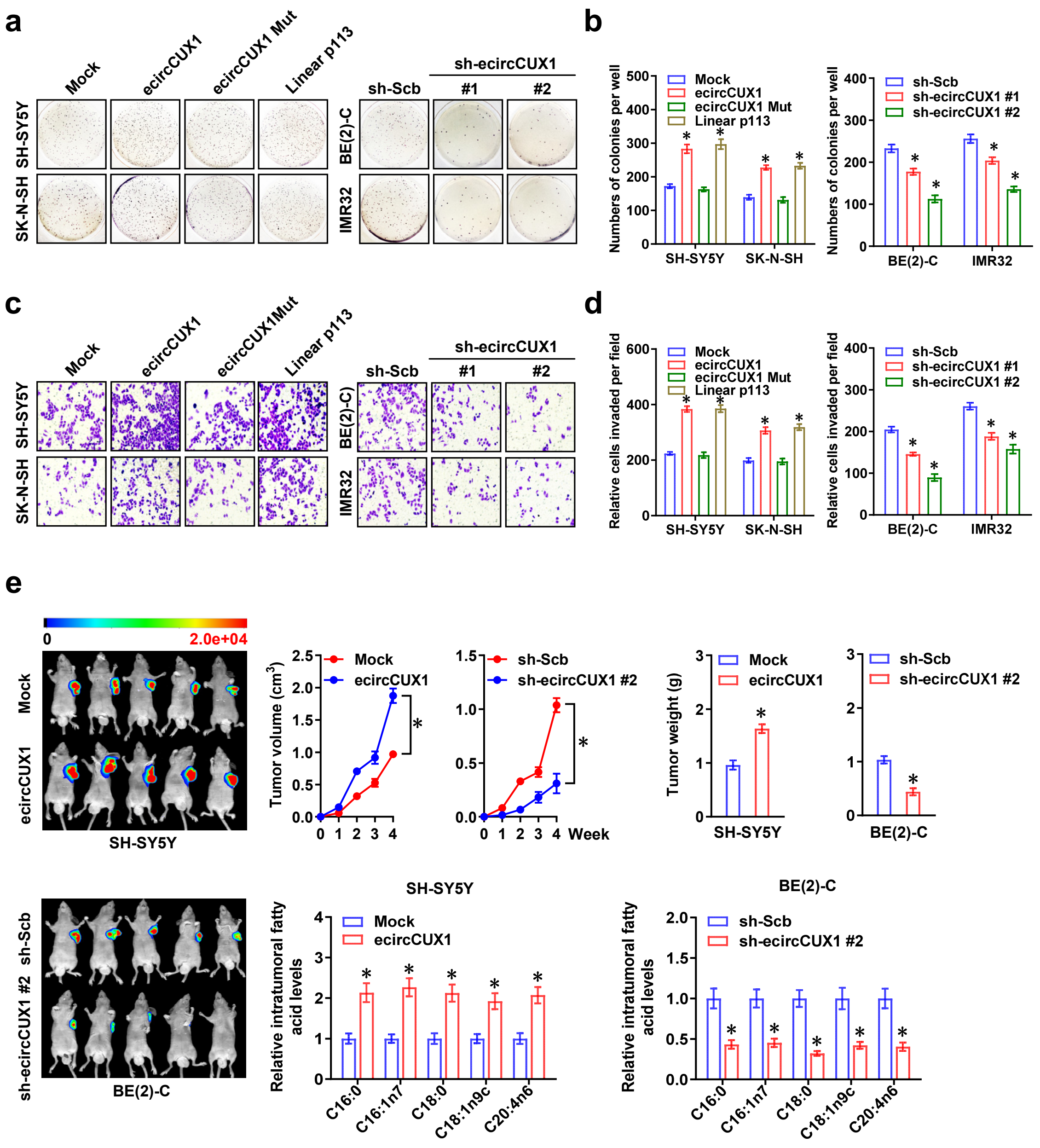

Supplementary Figure S4. ***ecircCUX1*** **promotes fatty acid oxidation and growth of NB via encoding p113. a-d** Representative images and quantification of soft agar (a and b) and matrigel invasion (c and d) assays indicating the anchorage-independent growth and invasion of NB cells stably transfected with empty vector (mock), *ecircCUX1*, ecircCUX1 with ORF mutation (*ecircCUX1* Mut), *p113*, scramble shRNA (sh-Scb), or sh-ecircCUX1. **e** *In vivo* imaging (left panel), growth curve (right upper panel), weight at the end points (right upper panel), and fatty acid levels (right lower panel) of xenografts in nude mice formed by subcutaneous injection of SH-SY5Y and BE(2)-C cells stably transfected with mock, *ecircCUX1*, sh-Scb, or sh-ecircCUX1 #2 (n=5 for each group). ANOVA compared the difference in **b**, **d** and **e**. *P<0.05 vs. mock or sh-Scb. Data are shown as mean ± s.e.m. (error bars) and representative of three independent experiments in **a**-**d**.

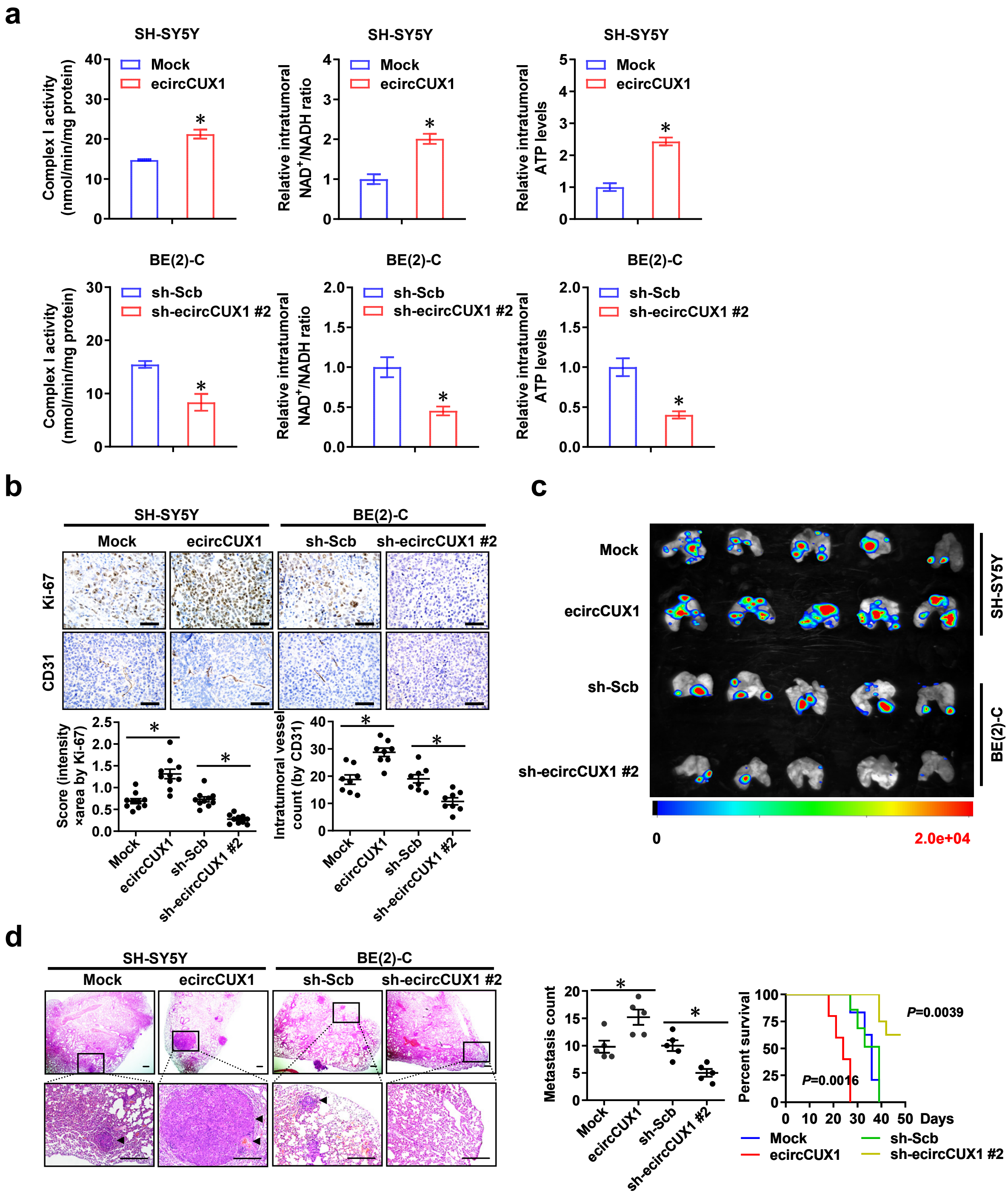

Supplementary Figure S5. *ecircCUX1* facilitates mitochondrial complex I activity and aggressiveness of NB *in vivo*. a Relative complex I activity, NAD^+/NADH ratio, ATP levels within xenografts in nude mice formed by subcutaneous injection of SH-SY5Y and BE(2)-C cells stably transfected with empty vector (mock), *ecircCUX1*, scramble shRNA (sh-Scb), or sh-ecircCUX1 #2 (*n*=5 for each group). **b** Representative images (upper panel) and quantification (lower panel) of immunohistochemical staining showing the expression of Ki-67 and CD31 within xenografts formed by subcutaneous injection of SH-SY5Y and BE(2)-C cells stably transfected with mock, *ecircCUX1*, sh-Scb, or sh-ecircCUX1 #2 (*n*=5 for each group). Scale bars: 50 μm. **c** and **d** Representative fluorescence images (c), hematoxylin & eosin staining (d, left panel), lung metastatic counts (d, right panel), and Kaplan–Meier curves (d, right panel) of nude mice treated with tail vein injection of SH-SY5Y and BE(2)-C cells stably transfected with mock, *ecircCUX1*, sh-Scb, or sh-ecircCUX1 #2 (*n*=5 for each group). Scale bars: 100 μm. Student's *t* test compared the difference in **a**, **b**, and **d**. Log-rank test for survival comparison in **d**. **P*< 0.05 vs. mock or sh-Scb. Data are shown as mean ± s.e.m. (error bars) and representative of three independent experiments in **a**.

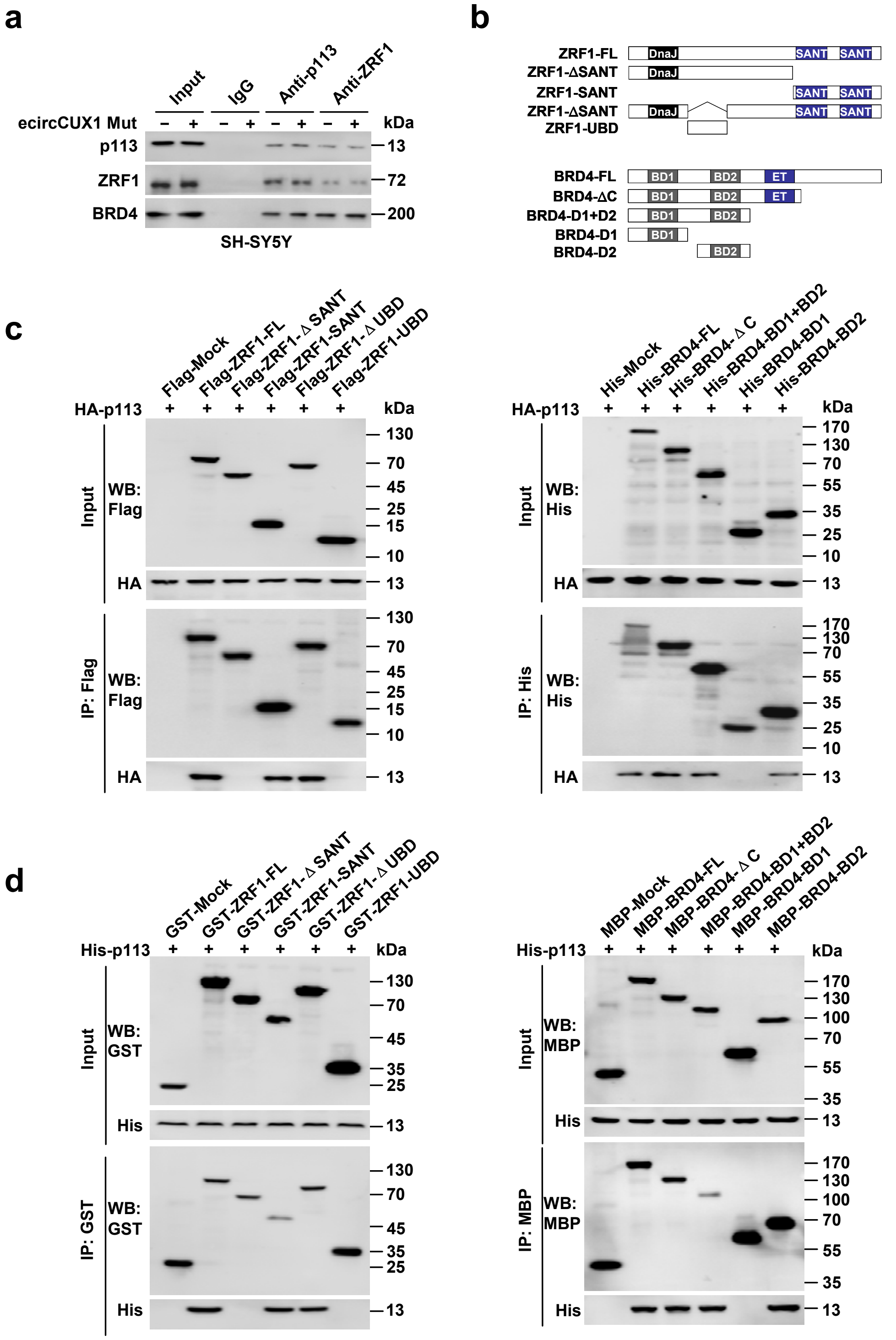

Supplementary Figure S6. Interaction of p113 with ZRF1 and BRD4 in NB cells. a Co-IP and western blot assays indicating the interaction among p113, ZRF1, and BRD4 in SH-SY5Y cells stably transfected with empty vector (mock) or *ecircCUX1* with ORF mutation (*ecircCUX1* Mut). The immunoglobulin G (IgG)-bound protein was taken as negative control. **b** Schematic illustration revealing domains of ZRF1 and BRD4 protein. **c** and **d** Co-IP and western blot assays showing the interaction of HA-tagged p113 with truncations of Flag-tagged ZRF1 or His-tagged BRD4, as well as His-tagged p113 with recombinant protein of GST-tagged ZRF1 or MBP-tagged BRD4. Data are representative of three independent experiments in **a**, **c** and **d**.

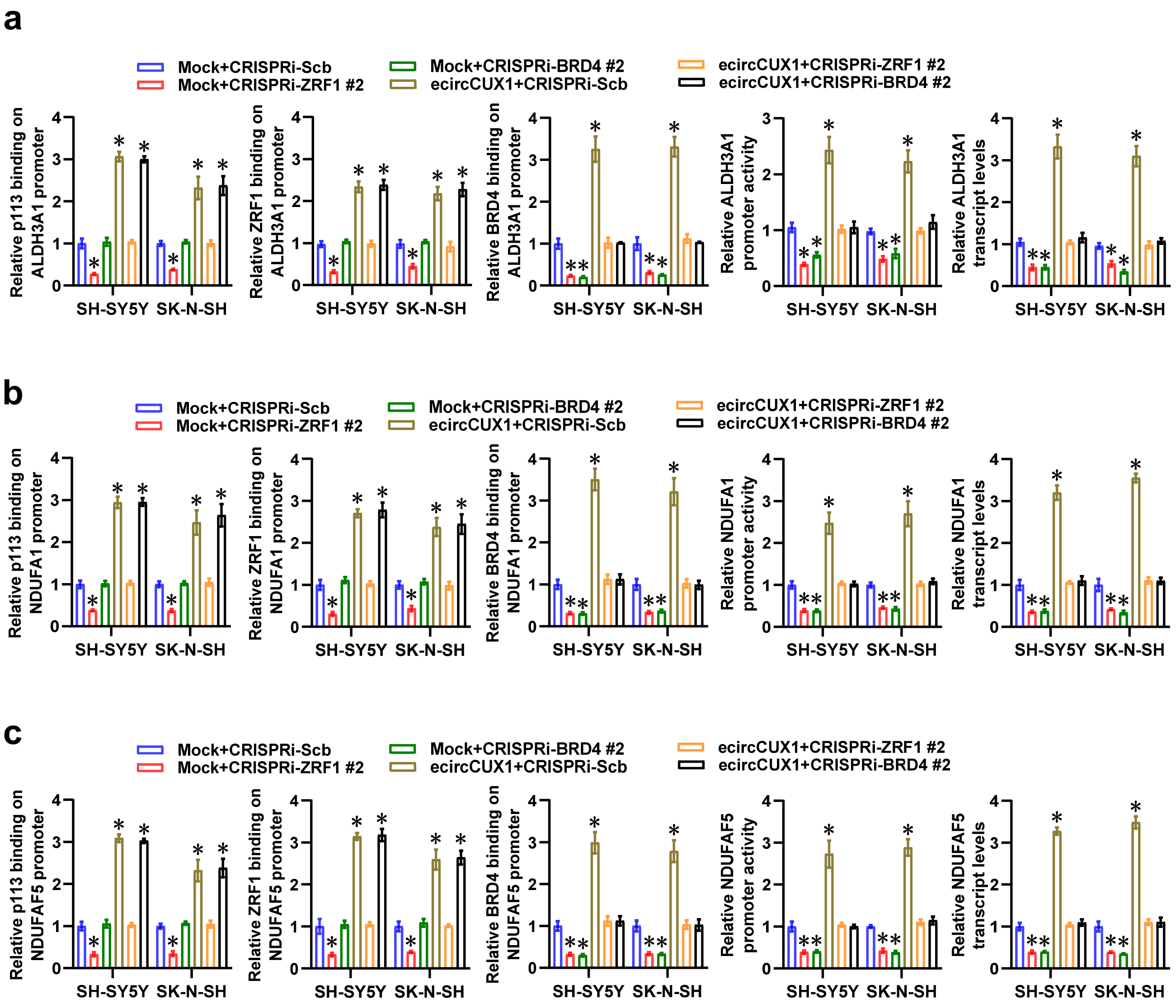

Supplementary Figure S7. ***ecircCUX1* facilitates expression of *ALDH3A1*, *NDUFA1*, or *NDUFAF5* in NB cells.** ChIP and qPCR (normalized to input, *n*=5), dual-luciferase (*n*=4), and real-time qRT-PCR (normalized to *β-actin*, *n*=4) assays indicating the enrichment of p113, ZRF1 and BRD4, promoter activity, and transcript levels of *ALDH3A1* (a), *NDUFA1* (b), or *NDUFAF5* (c) in SH-SY5Y and SK-N-SH cells stably transfected with empty vector (mock) or *ecircCUX1*, and those co-transfected with sgRNA specific against *ZRF1* or *BRD4* for CRISPRi. ANOVA compared the difference in **a**-**c**. **P*<0.05 vs. mock+CRISPRi-Scb. Data are shown as mean ± s.e.m. (error bars) and representative of three independent experiments in **a**-**c**.

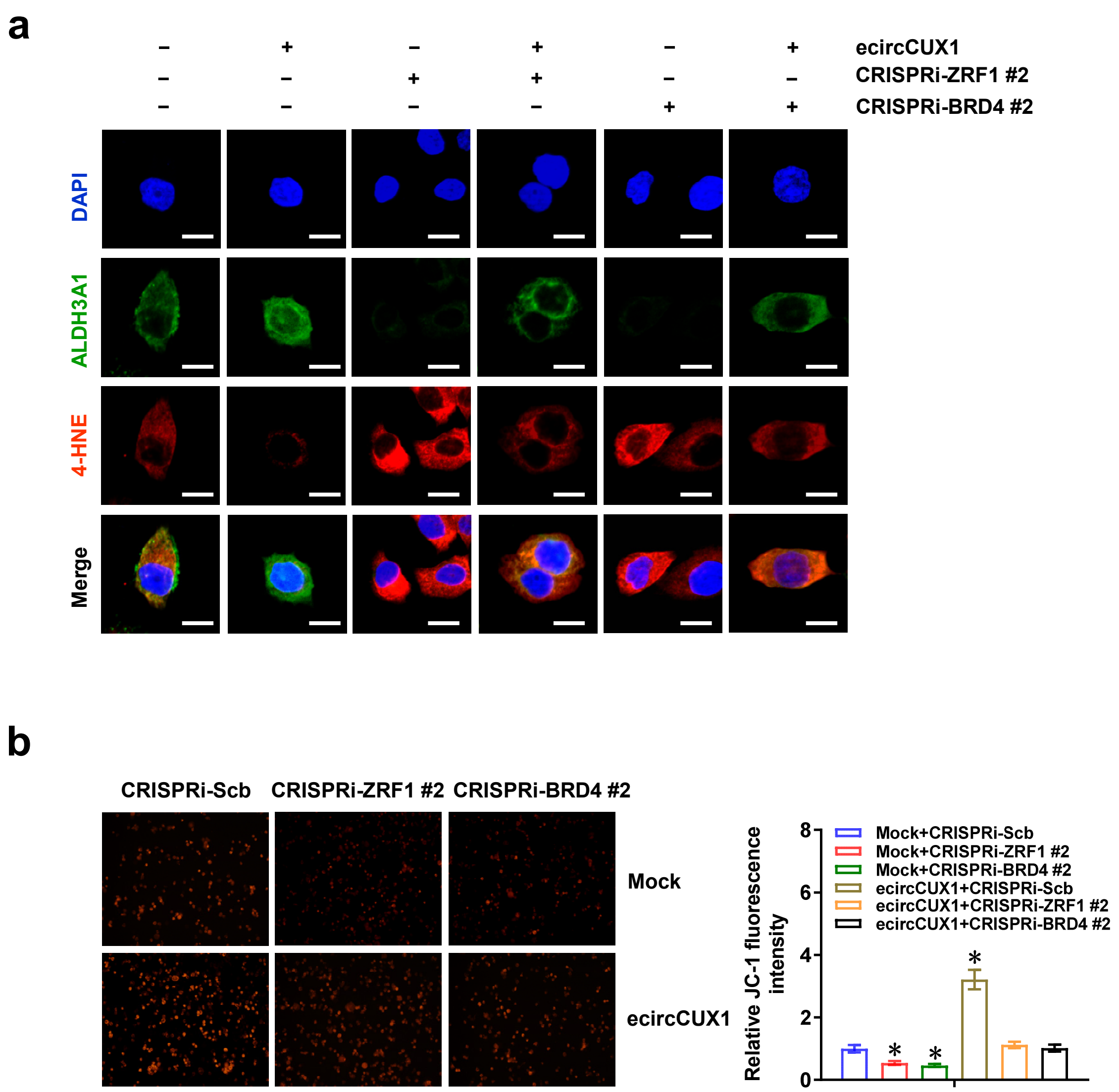

Supplementary Figure S8. ***ecircCUX1* reduces peroxidated lipids and increases mitochondrial membrane potential via ZRF1 or BRD4 in NB cells. a** Representative confocal images showing the expression of ALDH3A1 and 4-HNE in SH-SY5Y cells stably transfected with empty vector (mock) or *ecircCUX1*, and those co-transfected with sgRNA specific against *ZRF1* or *BRD4* for CRISPRi. **b** Representative images (left panel) and quantification (right panel) of JC-1 staining in SH-SY5Y cells stably transfected with mock or *ecircCUX1*, and those co-transfected with sgRNA specific against *ZRF1* or *BRD4* for CRISPRi. ANOVA compared the difference in **b**. *P<0.05 vs. mock+CRISPRi-Scb. Data are shown as mean ± s.e.m. (error bars) and representative of three independent experiments in **a** and **b**.

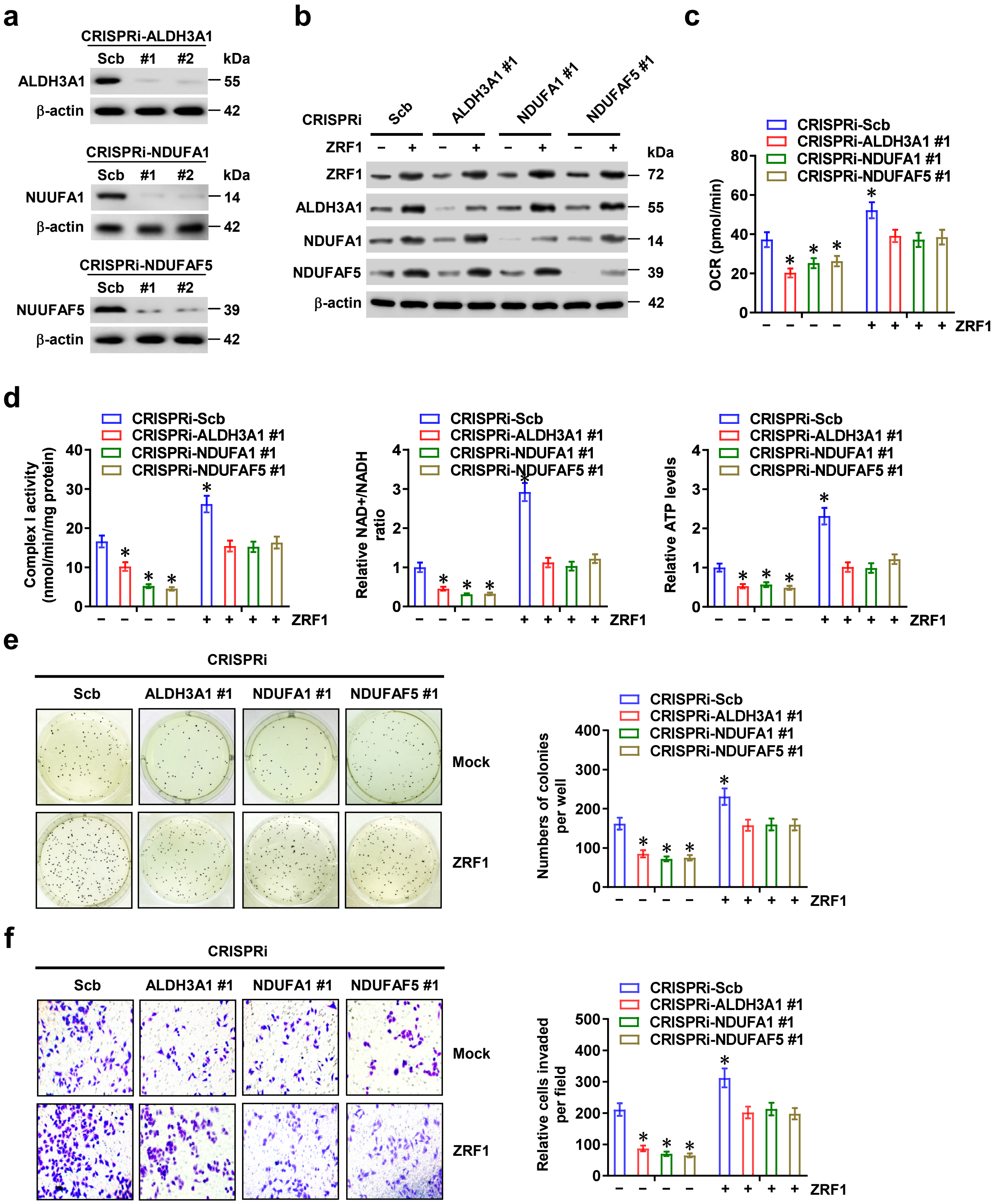

Supplementary Figure S9. *ZRF1* promotes mitochondrial complex I activity, growth, and aggressiveness of NB cells via target genes. a and **b** Western blot assay showing the levels of ZRF1, ALDH3A1, NDUFA1, or NDUFAF5 in BE(2)-C cells stably transfected with sgRNA specific against *ALDH3A1*, *NDUFA1*, or *NDUFAF5* for CRISPRi, and those co-transfected with empty vector (mock) or *ZRF1*. **c** and **d** Relative OCR levels (c), complex I activity, NAD^+/NADH ratio, ATP levels (d) in BE(2)-C cells stably transfected with mock or *ZRF1*, and those co-transfected with sgRNA specific against *ALDH3A1*, *NDUFA1*, or *NDUFAF5* for CRISPRi. **e** and **f** Representative images (left panel) and quantification (right panel) of soft agar (e) and matrigel invasion (f) assays indicating the anchorage-independent growth and invasion of BE(2)-C cells stably transfected with mock or *ZRF1*, and those co-transfected with sgRNA specific against *ALDH3A1*, *NDUFA1*, or *NDUFAF5* for CRISPRi. ANOVA compared the difference in **c**-**f**. *$P<0.05$ vs. mock+CRISPRi-Scb. Data are shown as mean ± s.e.m. (error bars) and representative of three independent experiments in **a**-**f**.

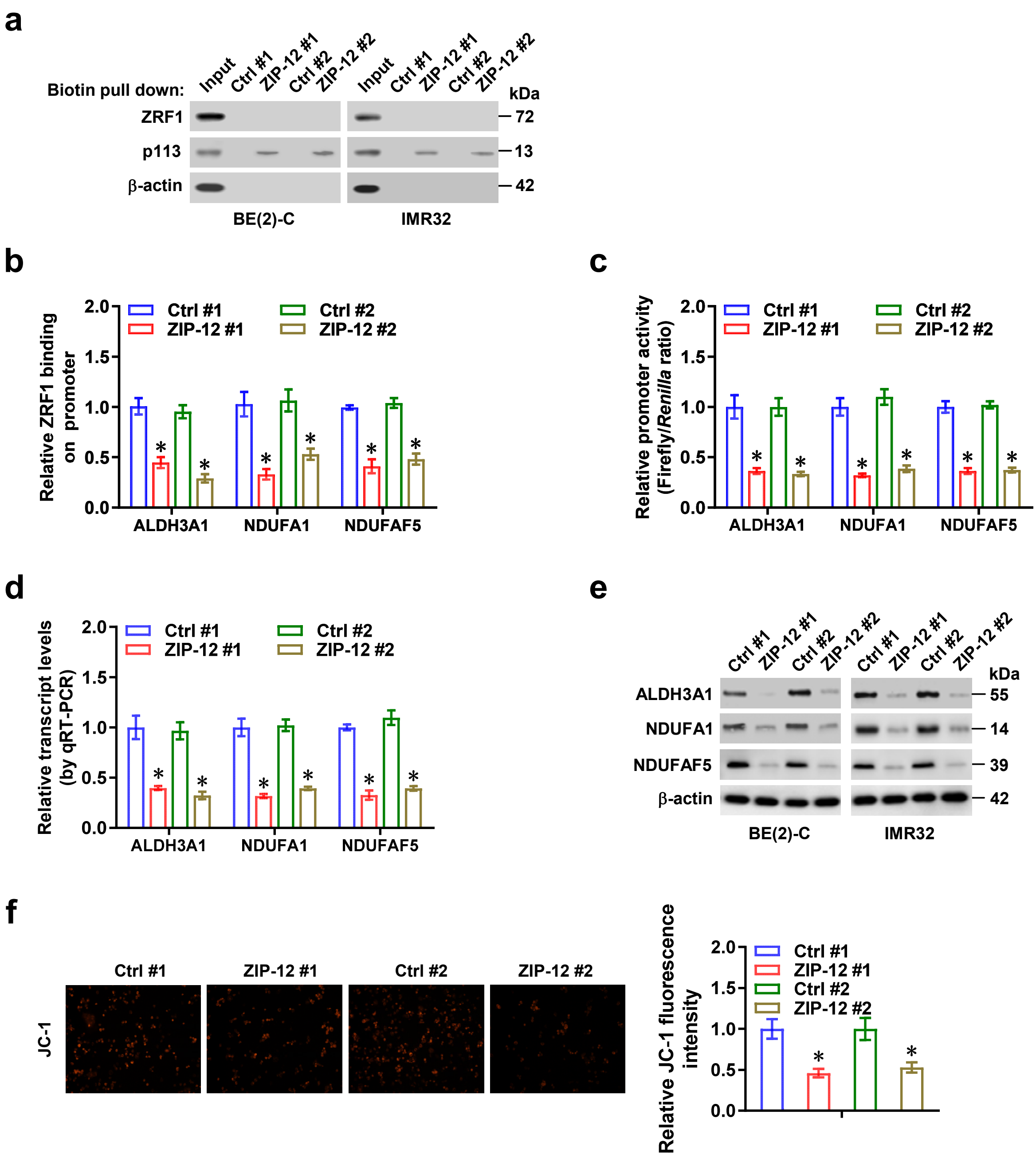

Supplementary Figure S10. Effects of inhibitory peptides blocking p113-ZRF1 interaction. a Biotin-labeled peptide pull-down and western blot assays showing the binding of mutant control (Ctrl) or ZIP-12 peptides (20 μmol·L^{-1}) to p113 within lysates of BE(2)-C and IMR32 cells. **b-e** ChIP and qPCR (b, normalized to input, *n*=5), dual-luciferase (c, *n*=4), real-time qRT-PCR (d, normalized to *β-actin*, *n*=4), and western blot (e) assays showing the ZRF1 enrichment, promoter activity, and expression levels of *ALDH3A1*, *NDUFA1*, or *NDUFAF5* in BE(2)-C cells treated with mutant control (Ctrl) or ZIP-12 peptides (20 μmol·L^{-1}) for 24 hrs. **f** Representative images (left panel) and quantification (right panel) of JC-1 staining in BE(2)-C cells treated with mutant control (Ctrl) or ZIP-12 peptides (20 μmol·L^{-1}) for 24 hrs. Student's *t* test compared the difference in **b-d** and **f**. **P*<0.05 vs. Ctrl. Data are shown as mean ± s.e.m. (error bars) and representative of three independent experiments in **a**-**f**.

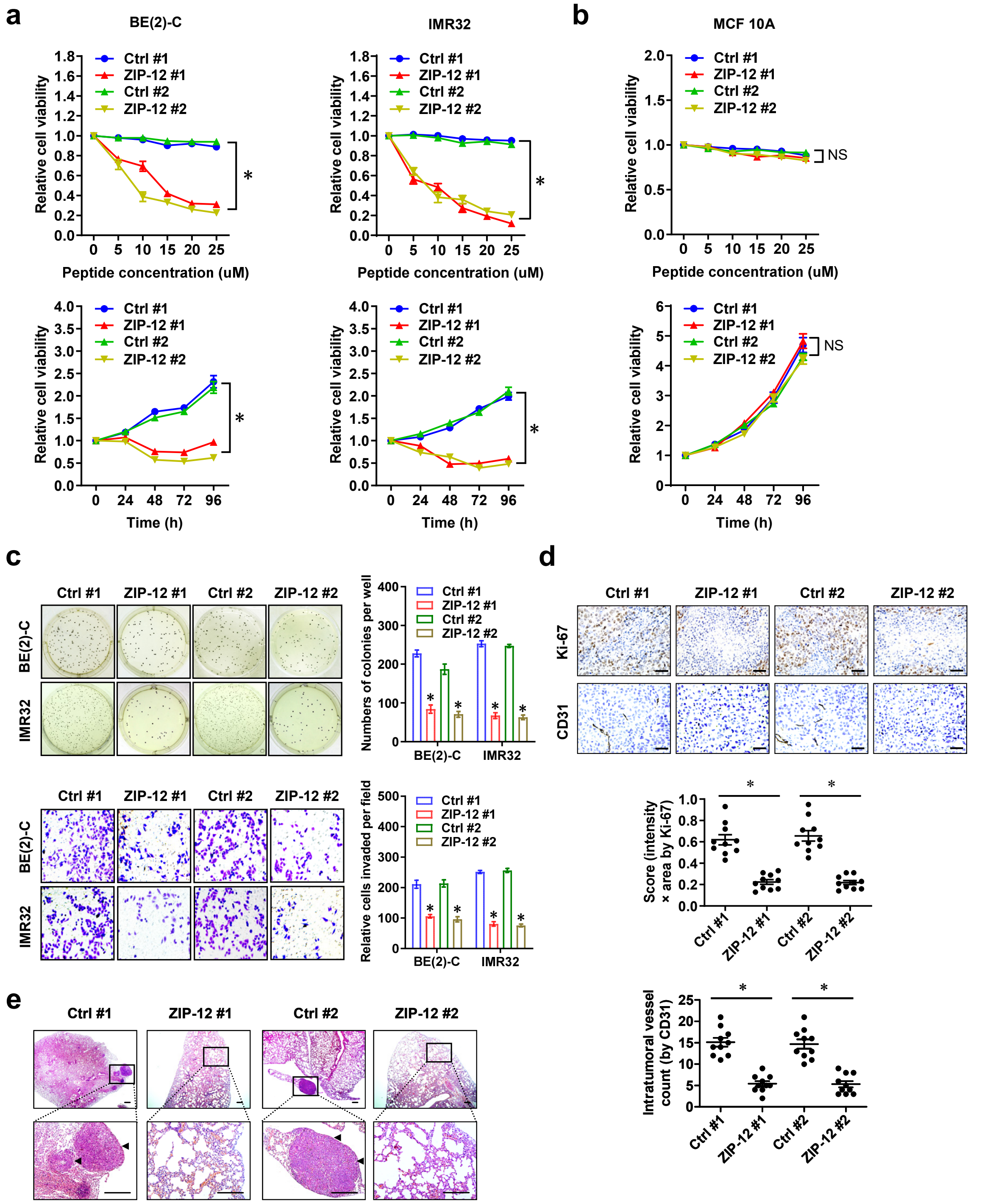

Supplementary Figure S11. ZIP-12 inhibits viabilities, growth, invasion, and metastasis of NB cells. **a** and **b** MTT colorimetric assay showing the viabilities of BE(2)-C, IMR32 (a), and MCF 10A (b) cells treated with mutant control (Ctrl) or ZIP-12 peptides as indicated. **c** Representative images (left panel) and quantification (right panel) of soft agar and matrigel invasion assays indicating the anchorage-independent growth and invasion of BE(2)-C and IMR-32 cells treated with Ctrl or ZIP-12 (20 $\mu mol \cdot L^{-1}$) for 48 hrs. **d** Representative images (upper panel) and quantification (lower panel) of immunohistochemical staining showing the expression of Ki-67 and CD31 within subcutaneous xenografts formed by injection of BE(2)-C cells in nude mice (*n*=5 per group) that were treated with intravenous injection of Ctrl or ZIP-12 peptides (5 $mg \cdot kg^{-1}$). **e** Hematoxylin & eosin staining indicating lung metastatic colonization of nude mice (*n*=5 for each group) treated with tail vein injection of BE(2)-C cells, Ctrl or ZIP-12 peptides (5 $mg \cdot kg^{-1}$). Scale bars: 100 μm. ANOVA and Student's *t* test compared the difference in **a-d**. *P<0.05 vs. Ctrl. Data are shown as mean ± s.e.m. (error bars) in **a-d**.

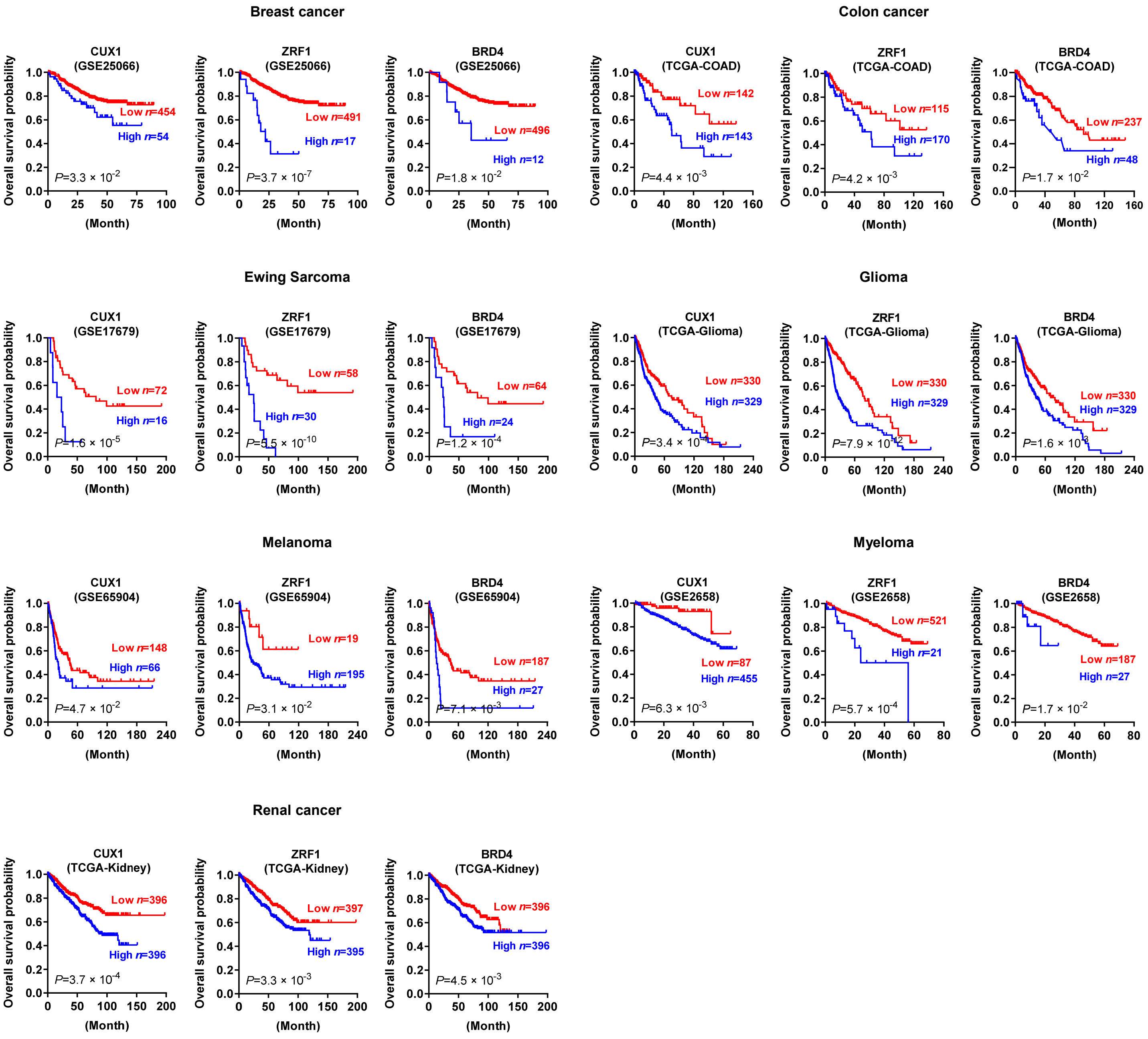

Supplementary Figure S12. Kaplan-Meier curves of *CUX1*, *ZRF1* and *BRD4* in multiple cancers. Kaplan-Meier curves indicating the survival of patients with low or high expression of *CUX1*, *ZRF1* or *BRD4* in breast cancer (cutoff values=7.196, 8.617, and 9.531), colon cancer (cutoff values=11.456, 10.152, and 11.104), Ewing sarcoma (cutoff values=7.962, 8.995, and 9.042), glioma (cutoff values=3.324, 2.471, and 3.201), melanoma (cutoff values=7.227, 7.379, and 7.416), myeloma (cutoff values=7.153, 9.059, and 9.381), or renal cancer (cutoff values=3.198, 2.448, and 3.021).

Supplementary Table S1 Primer sets used for RT-PCR, qPCR and ChIP

Primer set	Primers	Sequence	Product size (bp)	Application
ecircCUX1 (Divergent)	Forward	5’-CGCTCCAGCCTAGAAGTTGAGT-3'	220	RT-PCR
	Reverse	5’-TGCCCTCTGGTTTGCCCTTTCA-3'		
ecircCUX1 (Convergent)	Forward	5’-CTCAGAGAGAGGCGGAGACC-3'	280	RT-PCR
	Reverse	5’-TTGAGTGTGCTGTTTTTGGC-3'		
β-actin (Convergent)	Forward	5'-TGCCCATCTACGAGGGGTATG-3’	156	RT-PCR qPCR
	Reverse	5'-TCTCCTTAATGTCACGCACGATTT-3’		
β-actin (Divergent)	Forward	5'-AAATCGTGCGTGACATTAAGGAGA-3’	-	RT-PCR
	Reverse	5'-CATACCCCTCGTAGATGGGCA-3’		
GAPDH	Forward	5'-AGAAGGCTGGGGCTCATTTG-3'	258	qPCR
	Reverse	5'-AGGGGCCATCCACAGTCTTC-3'		
U1	Forward	5'-ACTTACCTGGCAGGGGAGATACC-3'	137	qPCR
	Reverse	5'-CCACTACCACAAATTATGCAGTCG-3'		
CUX1	Forward	5'-CCAGAGCCTGAACAGACTATTT-3'	283	qPCR
	Reverse	5'-CTTTAAGGCAGGGTCGAGGGCA-3'		
ecircCUX1 Flag KI	Forward	5'-CTTGGCAGGACATGCTGTATTT-3'	667	PCR
	Reverse	5'-CTGTCATGGATGCCACAGCTAC-3'		
ALDH3A1	Forward	5'-CCTGCACAAGAATGAATGGA-3'	210	qPCR
	Reverse	5'-GTGAGGTTGAAGGGGTAGTT-3'		
NDUFA1	Forward	5'-TTCGAGATTCTCCCCGGACT-3'	182	qPCR
	Reverse	5'-GACACATAGTAACGATCAAC-3'		
NDUFAF5	Forward	5'-ACCTGGGACATCTGCTTGGG-3'	159	qPCR
	Reverse	5'-CTCGATGCAGCAGGGCTTTT-3'		
ALDH3A1 (-333/-151)	Forward	5'-GCAAGTCTGGAAAGCTGGAAGA-3'	183	ChIP
	Reverse	5'-CCCAGGTTTTGGGGATTAGGTC-3'		
NDUFA1 (-648/-388)	Forward	5'-GCTGCGCTCAAAGATGGCTTGT-3'	261	ChIP
	Reverse	5'-TGCACTGTGGTTCGACCGGAAA-3'		
NDUFAF5 (-333/-63)	Forward	5'-AAGACTGAAGAATGAGACCGAA-3'	271	ChIP
	Reverse	5'-AGGGAACGTGCAAGTAGTTTGG-3'		

CUX1, cut like homeobox 1; ecircCUX1, exonic circular RNA derived from CUX1; GAPDH, glyceraldehyde 3-phosphate dehydrogenase; KI, Knock in; ALDH3A1, aldehyde dehydrogenase 3 family member A1; NDUFA1, NADH:ubiquinone oxidoreductase subunit A1; NDUFAF5, NADH:ubiquinone oxidoreductase complex assembly factor 5; RT-PCR: reverse transcription PCR; qPCR: quantitative PCR; ChIP, chromatin immunoprecipitation.

Supplementary Table S2 Oligonucleotide sets used for constructs

Primer Set	Sequences
pLenti-ecircCUX1	5'-CCGGAATTCTGAAATATGCTATCTTACAGGGCCGACGAGATTGAAATGA-3' (sense);<br>5'-CGCGGATCCTCAAGAAAAAATATATTCACTTTGAGTGTGCTGTTTTTGG-3' (antisense)
pcDNA3.1-p113	5'-CGCGGATCCCAAAGTGCTGAGATTACAGGCGT-3' (sense);<br>5'-CTAGCTAGCTGCTGGGATTACAGGTGTGAGCT-3' (antisense)
ecircCUX1-3Flag	5'-GGACTACAAAGATGATGACGATAAATGAAATGATCATGACGGACCTTG-3' (sense);<br>5'-CTTATCGTCGTCATCCTTGTAATCATCTCGTCGGCCCTGGTACCGCC-3' (antisense)
ecircCUX1 Mut 1	5'-CACACTCAAACCAAGAAGCAAGGAAAAGAATTAGGCTCGGCAC-3' (sense);<br>5'-CCTTGCTTCTTGGTTTGAGTGTGCTGTTTTTGGCGCTCAGCTG-3' (antisense)
ecircCUX1 Mut 2	5’-GCACACTCAACAGTAAGAAGCAAGGAAAAGAATTAGGCTCGG-3' (sense);<br>5’-TGCTTCTTACTGTTGAGTGTGCTGTTTTTGGCGCTCAGCTGC-3' (antisense)
ecircCUX1 Mut 3	5’-TAAATGAAATCATCGTGACGGACCTTGAAAGGGCAAACCAGAGGGC-3' (sense);<br>5’-AAGGTCCGTCACGATGATTTCATTTATCGTCATCATCTTTGTAGTC-3' (antisense)
ecircCUX1 Mut 4	5’-GATCATGACGTAGCTTGAAAGGGCAAACCAGAGGGCAGAGGTGG-3' (sense);<br>5’-GCCCTTTCAAGCTACGTCATGATCATTTCATTTATCGTCATCAT-3' (antisense)
ecircCUX1-IRES-1	5’-TTCTCAAAAATGAACAATAAGGATCCGAGTTGGCCGCCAAGGAGCGGG-3' (sense);<br>5’-ATGTTTTTGGCGTCTTCCATGAATTCTGCTGTTTTTGGCGCTCAGCTG-3’ (antisense)
ecircCUX1-IRES-2	5’-TTCTCAAAAATGAACAATAAGGATCCGGAGGACGTGCAGAGACTCCAG-3' (sense);<br>5’-ATGTTTTTGGCGTCTTCCATGAATTCTGAGTGTGCTGTTTTTGGCGCT-3’ (antisense)
p-Luc2-IRES-Reporter	5’-GAATTCATGGAAGACGCCAAAAACAT-3' (sense);<br>5’-GGATCCTTATTGTTCATTTTTGAGAA-3' (antisense)
IGF1R IRES	5'-CGCGGATCCCCGCCTTCGGAGTATTGTTTCC-3' (sense);<br>5'-CCGGAATTCTCCTTTTATTTGGGATGAAATTCCC-3' (antisense)
pLenti-ZRF1	5'-CTTGGGCTGCAGGTCGACTCTAGAGGATCCATGCTGCTTCTGCCAAGCGCCGC-3' (sense);<br>5'-GTCATCGTCATCCTTGTAGTCCATACCGGTTTTCTTGGCTCTACTTGCATTCA-3' (antisense)
pLenti-BRD4	5'-CTTGGGCTGCAGGTCGACTCTAGAGGATCCATGTCTGCGGAGAGCGGCCCTGG-3' (sense);<br>5'-GTCATCGTCATCCTTGTAGTCCATACCGGTGAAAAGATTTTCTTCAAATATTG-3' (antisense)
pGL3-ZRF1 luc	5'-CGTCAAGCATAGTCAAGCTATGTCAAGCATAGTCAAGCA-3' (sense);<br>5'-AGCTTGCTTGACTATGCTTGACATAGCTTGACTATGCTTGACGGTAC-3' (antisense)
pBiFC-VC155-p113	5'-ATGGCCATGGAGGCCCGAATTCGGATGATCATGACGGACCTTGAAAG-3' (sense);<br>5’-TTTTGCACGCCGGACGGGTACCATCTCGTCGGCCTTTGAGTG-3’ (antisense)
pBiFC-VC155-ZRF1	5'-ATGGCCATGGAGGCCCGAATTCGGATGCTGCTTCTGCCAAGCGCCGC-3' (sense);<br>5’-TTTTGCACGCCGGACGGGTACCTTTCTTGGCTCTACTTGCATTCA-3’ (antisense)
pBiFC-VN173-ZRF1	5'-ACAAGCTTGCGGCCGCGAATTCCATGCTGCTTCTGCCAAGCGCCGC-3' (sense);<br>5’-TCTTCTAGAGTCGACTGGTACCCCTTTCTTGGCTCTACTTGCATTCA-3’ (antisense)
pBiFC-VN173-BRD4	5'-ACAAGCTTGCGGCCGCGAATTCCATGTCTGCGGAGAGCGGCCCTGGGA-3' (sense);<br>5’-TCTTCTAGAGTCGACTGGTACCCCGAAAAGATTTTCTTCAAATATTGAC-3’ (antisense)
pCMV-HA-p113	5'-GCCGCTCGAGCCGCCACCATGATCATGACGGACCTTGAAA-3' (sense);<br>5’-ATTTGCGGCCGCATCTCGTCGGCCTTTGAGTGTGCTGTTTTTGGCG-3’ (antisense)
pET-28A-p113	5'-CGCGGATCCATGATCATGACGGACCTTGAAA-3' (sense);<br>5'-GCCGCTCGAGATCTCGTCGGCCTTTGAGTGTG-3’ (antisense)
pCMV-3Tag-1A-ZRF1-FL	5'-CGCGGATCCATGCTGCTTCTGCCAAGCGCCGC-3' (sense);<br>5'-GCCGCTCGAGTTTCTTGGCTCTACTTGCATTCA-3’ (antisense)
pCMV-3Tag-1A-ZRF1-ΔSANT	5'-CGCGGATCCATGCTGCTTCTGCCAAGCGCCGC-3' (sense);<br>5'-GCCGCTCGAGATTTCCACCTCCACCAGTTGATT-3’ (antisense)
pCMV-3Tag-1A-ZRF1-SANT	5'-CGCGGATCCGGAAGTAAAAATTGGTCAGAAGA-3' (sense);<br>5'-GCCGCTCGAGTTTCTTGGCTCTACTTGCATTCA-3’ (antisense)
pCMV-3Tag-1A-ZRF1-ΔUBD-1	5'-CGCGGATCCAACAGAGCAACAAGAGCACAAA-3' (sense);<br>5'- ACTGTTAAATGCTCGTCTTTTCA-3’ (antisense)
pCMV-3Tag-1A-ZRF1-ΔUBD-2	5'-CGCGGATCCGTAGATCCTACTTTTGATAACTC-3' (sense);<br>5'-GCCGCTCGAGTTTCTTGGCTCTACTTGCATTCA-3’ (antisense)
pCMV-3Tag-1A-ZRF1-UBD	5'-CGCGGATCCGTAGATCCTACTTTTGATAACTC-3' (sense);<br>5'-GCCGCTCGAGCTGCTTTTCAATCCATCTCCTCT-3’ (antisense)
pCDNA4-His-BRD4-ΔC	5'-CGCGGATCCATGTCTGCGGAGAGCGGCCCTGG-3’ (sense);<br>5'-GCCGCTCGAGGTGCCCCTTCTTTTTTGACTTCG-3' (antisense)
pCDNA4-His-BRD4-BD1+BD2	5'-CGCGGATCCATGTCTGCGGAGAGCGGCCCTGG-3’ (sense);<br>5'-GCCGCTCGAGGGAGGACACGGCCACCACTGGCT-3' (antisense)
pCDNA4-His-BRD4-BD1	5'-CGCGGATCCATGTCTGCGGAGAGCGGCCCTGG-3’ (sense);<br>5'-GCCGCTCGAGAGTTGTGTTTGGTACCGTGGAAA-3' (antisense)
pCDNA4-His-BRD4-BD2	5'-CGCGGATCCCCCCCCGCCAGTGCCCCCCCAGCC-3’ (sense);<br>5'-GCCGCTCGAGGGAGGACACGGCCACCACTGGCT-3' (antisense)
pGEX-6P-1-ZRF1-FL	5'-CGCGGATCCATGCTGCTTCTGCCAAGCGCCGC-3' (sense);<br>5'-GCCGCTCGAGTTTCTTGGCTCTACTTGCATTCA-3’ (antisense)
pGEX-6P-1-ZRF1-ΔSANT	5'-CGCGGATCCATGCTGCTTCTGCCAAGCGCCGC-3' (sense);<br>5'-GCCGCTCGAGATTTCCACCTCCACCAGTTGATT-3’ (antisense)
pGEX-6P-1-ZRF1-SANT	5'-CGCGGATCCGGAAGTAAAAATTGGTCAGAAGA-3' (sense);<br>5'-GCCGCTCGAGTTTCTTGGCTCTACTTGCATTCA-3’ (antisense)
pGEX-6P-1-ZRF1-ΔUBD-1	5'-CGCGGATCCATGCTGCTTCTGCCAAGCGCCGC-3' (sense);<br>5'- ACTGTTAAATGCTCGTCTTTTCA-3’ (antisense)
pGEX-6P-1-ZRF1-ΔUBD-2	5'-CGCGGATCCGTAGATCCTACTTTTGATAACTC-3' (sense);<br>5'-GCCGCTCGAGTTTCTTGGCTCTACTTGCATTCA-3’ (antisense)
pGEX-6P-1-ZRF1-UBD	5'-CGCGGATCCGTAGATCCTACTTTTGATAACTC-3' (sense);<br>5'-GCCGCTCGAGCTGCTTTTCAATCCATCTCCTCT-3’ (antisense)

pMAL-c4X-BRD4-FL	5'-CGCGGATCCATGTCTGCGGAGAGCGGCCCTGG-3’ (sense);<br>5’-CCCAAGCTTGAAAAGATTTTCTTCAAATATTG-3' (antisense)
pMAL-c4X-BRD4-ΔC	5'-CGCGGATCCATGTCTGCGGAGAGCGGCCCTGG-3’ (sense);<br>5’-CCCAAGCTTGTGCCCCTTCTTTTTTGACTTCG-3' (antisense)
pMAL-c4X-BRD4-BD1+BD2	5'-CGCGGATCCATGTCTGCGGAGAGCGGCCCTGG-3’ (sense);<br>5’-CCCAAGCTTGGAGGACACGGCCACCACTGGCT-3' (antisense)
pMAL-c4X-BRD4-BD1	5'-CGCGGATCCATGTCTGCGGAGAGCGGCCCTGG-3’ (sense);<br>5’-CCCAAGCTTAGTTGTGTTTGGTACCGTGGAAA-3' (antisense)
pMAL-c4X-BRD4-BD2	5'-CGCGGATCCCCCCCCGCCAGTGCCCCCCCAGCC-3’ (sense);<br>5’-CCCAAGCTTGGAGGACACGGCCACCACTGGCT-3' (antisense)
pGL3-ALDH3A1-promoter	5'-CTAGCTAGCTGGAGGAAGACGACAGCATTGTGC-3' (sense);<br>5’-CCCAAGCTTAGAGCTGCCAGAGGTCCAGGAGGA-3' (antisense)
pGL3-NDUFA1-promoter	5'-CTAGCTAGCGGCGACAGATGAAACACTTGAATG-3' (sense);<br>5’-CCCAAGCTTCAAAACCCTACTTCTGGGTCCTTG-3' (antisense)
pGL3-NDUFAF5-promoter	5'-CTAGCTAGCAAGTCTCCAATGGTTTAGCATCTC-3' (sense);<br>5’-CCCAAGCTTACCTCCTCCTTCAGGTAGTCAAAT-3' (antisense)

ecircCUX1, exonic circular RNA derived from CUX1; Mut, mutation; IRES, internal ribosome entry site; IGF1R, insulin like growth factor 1 receptor; BiFC, bimolecular fluorescence complementation; ZRF1, Zuotin-related factor 1; BRD4, bromodomain-containing protein 4; ALDH3A1, aldehyde dehydrogenase 3 family member A1; NDUFA1, NADH:ubiquinone oxidoreductase subunit A1; NDUFAF5, NADH:ubiquinone oxidoreductase complex assembly factor 5.

Supplementary Table S3 Oligonucleotide sets used for short hairpin RNAs and CRISPR-Cas9/dCas9

Oligo Set	Sequences	
sh-Scb	Sense	5'-CCGGGCGAACGATCGAGTAAACGGACTCGAGTCCGTTTACTCGATCGTTCGCTTTTT-3'
	Antisense	5'-AATTCAAAAAGCGAACGATCGAGTAAACGGACTCGAGTCCGTTTACTCGATCGTTCGC-3'
sh-ecircCUX1 #1	Sense	5'-CCGGTGCACACTCAAAGGCCGACGCTCGAGCGTCGGCCTTTGAGTGTGCTTTTTG-3'
	Antisense	5'-GATCCAAAAAGCACACTCAAAGGCCGACGCTCGAGCGTCGGCCTTTGAGTGTGCA-3'
sh-ecircCUX1 #2	Sense	5'-CCGGTCAGCACACTCAAAGGCCGACTCGAGTCGGCCTTTGAGTGTGCTGTTTTTG-3'
	Antisense	5'-GATCCAAAAACAGCACACTCAAAGGCCGACTCGAGTCGGCCTTTGAGTGTGCTGA-3'
sgRNA-KI-p113	Sense	5'-CACCGCATGATCATTTCAATCTCGT-3'
	Antisense	5'-AAACACGAGATTGAAATGATCATGC-3'
sgRNA-CRISPRi-ZRF1 #1	Sense	5'-CACCGAGGTTACCGCACACGTTGGC-3'
	Antisense	5'-AAACGCCAACGTGTGCGGTAACCTC-3'
sgRNA-CRISPRi-ZRF1 #2	Sense	5'-CACCGCTTCCGGGATGGATCTTTCG-3'
	Antisense	5'-AAACCGAAAGATCCATCCCGGAAGC-3'
sgRNA-CRISPRi-BRD4 #1	Sense	5'-CACCGCCGAGGAGCCGAAGCAGTGG-3'
	Antisense	5'-AAACCCACTGCTTCGGCTCCTCGGC-3'
sgRNA-CRISPRi-BRD4 #2	Sense	5'-CACCGGTTCTGGTCTCCCGCAGCCG-3'
	Antisense	5'-AAACCGGCTGCGGGAGACCAGAACC-3'
sgRNA-CRISPRi-ALDH3A1 #1	Sense	5'-CACCGTAAATACGTCCCCTCTTGGC-3'
	Antisense	5'-AAACGCCAAGAGGGGACGTATTTAC-3'
sgRNA-CRISPRi-ALDH3A1 #2	Sense	5'-CACCGGATGGGCCGTCAGACTCCAT-3'
	Antisense	5'-AAACATGGAGTCTGACGGCCCATCC-3'
sgRNA-CRISPRi-NDUFA1 #1	Sense	5'-CACCGTGGCTACTGCGTACATCCAC-3'
	Antisense	5'-AAACGTGGATGTACGCAGTAGCCAC-3'
sgRNA-CRISPRi-NDUFA1 #2	Sense	5’-CACCGGGTAAGCCGGCTTCGGCCCG-3’
	Antisense	5’-AAACCGGGCCGAAGCCGGCTTACCC-3’
sgRNA-CRISPRi-NDUFAF5 #1	Sense	5’-CACCGAGTAGACACAAAAGCCGCGC-3’
	Antisense	5’-AAACGCGCGGCTTTTGTGTCTACTC-3’
sgRNA-CRISPRi-NDUFAF5 #2	Sense	5'-CACCGGGCGCTTATGTCGGCGACCT-3'
	Antisense	5'-AAACAGGTCGCCGACATAAGCGCCC-3'

ecircCUX1, exonic circular RNA derived from CUX1; ZRF1, Zuotin-related factor 1; BRD4, bromodomain-containing protein 4; ALDH3A1, aldehyde dehydrogenase 3 family member A1; NDUFA1, NADH:ubiquinone oxidoreductase subunit A1; NDUFAF5, NADH:ubiquinone oxidoreductase complex assembly factor 5; sgRNA, small guide RNA; KI, knock in; CRISPR, Clustered regularly interspaced short palindromic repeats; CRISPRi, CRISPR-dCas9-mediated interference; dCas9, dead mutant of Cas9 endonuclease; shRNA, short hairpin RNA.

Supplementary Table S4 Mass spectrometry analysis of proteins altered by serum deprivation

SH-SY5Y							SK-N-BE(2)		
ABCF1	CHCHD5	FAM192A	HNRPUL1	NAT10	PSMD12	TBCA	ABHD12	ISOC1	SLC5A6
ABCF2	CHCHD8	FAM207A	HOMER1	NBLA10388	PSMD3	TCF3	ALB	ITGB6	SLC7A1
ACP1	CHEK2	FAM20B	HOXC4	NCAM1	PSME3	TFB1M	ANAPC10	JAGN1	SLIT3
ACSS1	CHMP1A	FAR1	HPRT1	NCAM2	PTGES3	TGFB1	ANO10	JMJD6	SMIM4
ADPRHL2	CHMP2A	FAU	HSP90AB2P	NCAPD2	PYGL	TIMM8B	APOE	KIF11	SQLE
AGFG1	CHMP4B	FDFT1	HSPA8	NDUFA4	RAB30	TMEM123	ARHG	KIF23	SRC
ALAS1	CHTOP	FEAT	HTATIP	NEFM	RABEPK	TMEM41B	ARPC4-TTLL3	LENG8	SSR3
ALB	CLASP1	FIS1	HYAL3	NIT1	RABL6	TMSB10	B3GNT6	LOC221955	STXBP2
ALG1	CLCN7	FKBP1A	HYPK	NOSTRIN	RANBP3	TMUB1	BCKDHB	LRP2	SUSD1
ALG9	CLK1	FMR1	IFI30	NSMCE4A	RBM19	TNPO2	BCL7B	LZTS2	SYAP1
AMFR	CMTR1	FN3KRP	IFT81	NTMT1	RBM6	TNPO3	BLOC1S2	MAGOH	SYNE3
ANKHD1	CNIH4	FNBP1L	IKBKAP	NUBP2	RBM7	TPM1	BLVRB	MAPK14	TAF10
ANP32B	COX17	FUBP1	IMPDH1	NUDCD1	REL	TPM3	BRAP	METTL25	TCF3
ANP32E	COX6A1	FUS	IMPDH2	NUDCD2	RER1	TRIAP1	C13ORF1	MG44	TCOF1
APOE	COX6B1	FYTTD1	IRAK1	NUDT4	RHBDD1	TRIP13	C8ORF33	MIF	TGFB1
APRT	CRKL	G3BP	IST1	NUTF2	RNF126	TRIP6I4	CCDC22	MOCS1	TM4SF2
ARF4	CSDA	GARS	ITGB6	OGT	RPIA	TRMT11	CCDC43	MPC2	TMEM115
ARHGEF7	CSRP2	GART	ITPR1	OSBPL3	RPL26	TRMT61B	CD46	MRS2	TMEM14C
ARL1	CTPS1	GET4	JMJD6	OXSR1	RPL29	TRNT1	CDC23	MXRA7	TMEM167A
ASAP1	CUL5	GFM1	KARS	PANK2	RPL31	TTF2	CENPT	NCAPG2	TMEM245
ASCC2	CUX1	GLO1	KATNAL2	PCBD	RPL35	TUBA1A	CENTG3	NCSTN	TOP3A
ATP8	CWC15	GOPC	KIAA1211	PCNA	RPL37	TUBA1B	CMBL	NET1	TOPBP1
ATP8A1	CXORF58	GPATCH8	KIF11	PCNT	RPS15	TUBA1C	CMC1	NSMCE4A	TRAM1
AURKB	CYP51A1	GPSM1	KNS2	PDCL3	RPS17	TUBB	COX6A1	NUDCD2	TRIP6I4
B2M	DAK	GSK3A	LACTB	PDLIM5	RPS19	TUBB2B	CSNK1G2	NUSAP1	UBE2C
BANF1	DBI	GSTM3	LANCL1	PFKL	RPS23	TUBB2C	CUX1	OTUD3	UBE2J1
BANP	DCK	GTF2A1	LDAH	PFKM	RPS24	TUBB8	DAK	PARP2	UNG
BAT3	DDT	HCG_23833	LDHB	PFKP	RPS29	TWF2	DCX	PDCD5	UQCC1
BCAT1	DDX19A	HEL107	LENG8	PFN1	RPS6KA1	UBE2A	DKFZP686G2045	PIBF1	VDAC3
BLVRB	DDX20	HEL2	LGMN	PHF8	RPS6KA3	UBE2L3	DNAH11	PIG59	VTA1
BRAP	DDX5	HEL-76	LSM6	PHGDH	RPS7	UBN2	EGLN1	PIH1D1	XPO6
BSG	DIS3	HEL-S-1	LTA4H	PHKB	RPSA	UBR5	ENPP1	POLR2D	YA61
BTF3	DKFZP434P232	HEL-S-103	LUC7L2	PHOX2A	RTCA	UBXN1	ESRP1	PON2	ZC3HAV1L
BTF3L4	DKFZP547M202	HEL-S-105	MAD1L1	PHYHIPL	S100A10	UCK2	F2	PPAT	ZFYVE16
C14ORF159	DKFZP686E01144	HEL-S-112	MAP2K2	PICALM	S100A13	UQCC2	FABP5	PPP4C	ZNF234
C19ORF53	DKFZP686G2045	HEL-S-133P	MCTS1	PIG59	SCAMP5	VDAC3	FAM114A2	PRC1	
CACFD1	DNAH11	HEL-S-19	MEA1	PIN1	SEC16A	VPS37A	FAM219A	PRIM1	
CACYBP	DNAH12	HEL-S-44	METTL25	PISD	SEC24B	VPS4A	FDX1	PTDSS2	
CAD	DNAJA2	HEL-S-49	MEX3A	PLD3	SERBP1	WDR55	FN3KRP	RAB29	
CAMK4	DSP	HEL-S-61	MGC3731	PLGRKT	SERPINB6	WFS1	HBA2	RAVER2	
CASC5	DUSP3	HEL-S-70	MGST2	PMVK	SET	WUGSC	HBD/HBB	REL	
CCDC43	DYNC1LI2	HEL-S-73	MIF	POP7	SF3B2	YWHAZ	HCG_23833	RIOK1	
CCDC72	EDARADD	HEL-S-87P	MORF4L2	PPP1R14B	SLC11A2	ZBTB33	HIST1H2AH	RPL26L1	
CCNT1	EEF1A1	HGH1	MP68	PPP4C	SLC9A3R2	ZCCHC17	HIST1H2BA	RPP25	
CCS	EEF1B2	HIP1R	MRFAP1	PQBP1	SNW1	ZFYVE16	HIST1H3A	RPS28	
CD151	EIF3S1	HIST1H2BA	MRPS33	PRC1	SPECC1L	ZNF234	HMGN4	S100A10	
CDC42	EIF4E	HK2	MSH3	PRIC295	SPG20	ZNF24	HNRNPA3	SERINC1	
CDK2AP1	EL52	HLA-A	MTG2	PRKCI	SRC	ZNF711	HNRPA1	SERPINE2	
CEBPB	ELOF1	HLA-B	MYH10	PROCR	STMN2	ZNF768	HOXC4	SF3B2	
CECR2	ENO1	HLA-C	MYH14	PRPS1	STRA6	ZNF830	HPCAL1	SIGMAR1	
CENPE	EPB41L2	HMGN4	MYH9	PSMA6	STX1A	ZUBR1	HSD17B8	SLC16A1	
CENPM	EWSR1	HN1	MYL12A	PSMC2	STXBP2		HSP90AB2P	SLC23A2	
CENPP	F2	HNRNPAB	MYL6	PSMC3	SUPT4H1		HTATIP	SLC39A10	
CENPU	FAM114A2	HNRNPDL	NACA	PSMC4	SYNCRIP		INSM2	SLC39A14	
CENTG3	FAM129B	HNRPA1	NAPG	PSMD10	TAF15		IRAK1	SLC44A1	

Supplementary Table S5 p113 expression in human NB tissues

Group	Total number	p113 expression				Positive rates (%)	*P*-Value
		−	+	++	+++		
Age							
<1 year	20	11	6	2	1	45.0	0.001
⩾1 year	22	3	5	6	8	86.4	
Differentiation							
Well differentiated	8	6	1	1	0	25.0	
Poorly differentiated	28	8	10	6	4	71.4	0.001
Undifferentiated	6	0	0	1	5	100.0	
MKI							
<200	17	13	2	2	0	23.6	0.002
>200	25	1	9	6	9	96.0	
INSS stages							
Stage 1-2	14	8	5	1	0	42.9	
Stage 3-4	20	4	4	3	9	80.0	0.003
Stage 4S	8	2	2	4	0	75.0	
MYCN amplification							
No	34	12	10	6	6	64.7	0.158
Yes	8	2	1	2	3	75.0	

MKI, mitosis karyorrhexis index; INSS, international neuroblastoma staging system.

Supplementary Table S6 Mass spectrometry analysis of proteins pulled down by p113 antibody

AASDHPPT	CBX8	DHCR7	FHL2	IDH3B	MRPL21	NUP88	PYCR1	SLC16A1	TOMM40	ZNF346
ABLIM1	CCDC12	DHX29	FKBP5	IGF2	MRPL38	NVL	PYCR2	SLC1A5	TOMM70A	ZNF461
ACAA2	CCDC47	DHX30	FKBP8	IGKV1-8	MRPL39	OSTC	PYGL	SLC25A1	TOPBP1	ZNF569
ACBD3	CCDC59	DHX33	FLG2	IGKV1D-13	MRPL40	OXSR1	RAB11FIP1	SLC25A10	TPI1	ZNF579
ACLY	CCDC86	DHX57	FLII	IKBKAP	MRPL41	P4HB	RAB14	SLC25A11	TPM1	ZNF592
ACOT9	CCT7	DHX8	FLJ00385	ILKAP	MRPL44	PA2G4	RAB1A	SLC25A13	TPM3	ZNF593
ACSL3	CCT8	DKFZp434E1119	FLJ10154	IMP3	MRPL47	PACSIN3	RAB1B	SLC2A1	TPM4	ZNF622
ACTN1	CD2BP2	DKFZp451G231	FLYWCH1	IMP4	MRPL48	PARD3	RAB2	SLC39A7	TPT1	ZNF668
ACTN4	CD59	DKFZp666L156	FN1	INADL	MRPL55	PC4	RAB34	SLIRP	TRIM21	ZNF771
ACTR2	CDC2	DKFZp686A111	FP972	IPO4	MRPS12	PCM1	RAI14	SLMAP	TRIM27	ZNF96
ACTR3	CDC2L2	DKFZp686D1968	FUBP1	IPO7	MRPS15	PDCD4	RALB	SLU7	TRIOBP	ZRANB2
ADH5	CDC42EP1	DKFZp686E1893	GADD45GIP1	IPO9	MRPS16	PDCD6IP	RANBP1	SMARCC	TRIP13	
ADM	CDC73	DKFZp686E1899	GART	IRF2BP2	MRPS23	PDIA3	RANBP5	SMARCD	TRIP6	
AFAP1	CDK9	DKFZp686E23276	GFM1	IRS4	MRPS25	PDS5A	RANGAP1	SMARCE1	TRMT112	
AGBL5	CDKN2AIP	DKFZp686G2045	GFPT1	ITIH2	MRPS26	PDS5B	RAP2C	SMC2	TSFM	
AGK	CENPB	DKFZp686J0330	GLS	ITPA	MRPS31	PEG10	RARS	SMC4	TSPO	
AHCY	CFI	DKFZp686L0869	GLYR1	ITPR3	MRPS6	PFKL	RASL11B	SMC5	TSR3	
AHSG	CGN	DKFZp686M1669	GMPS	JUP	MSH2	PFN1	RBFOX1	SMPD4	TUFT1	
AIF1L	CHCHD1	DKFZp762I166	GNAI2	KANK2	MSH6	PFN2	RBM12B	SMU1	TWF1	
AKAP2	CHMP1A	DKFZp779I1858	GNAI3	KDELR2	MSL1	PGAM1	RBM15B	SNW1	TXLNG	
AKAP8L	CHMP2A	DNAJB1	GNAS	KIAA0020	MST065	PHF12	RBM19	SP1	UACA	
ALDH18A1	CHMP2B	DNAJB6	GNB1	KIAA0992	MTA2	PHF3	RBM22	SPATA5	UBAP2	
ALDH1B1	CHMP3	DNAJC11	GNB2	KIAA1671	MTCH2	PHGDH	RBM7	SPECC1	UBE1	
ALDH9A1	CHMP4B	DNAJC2	GNB4	KIF14	MTDH	PIG60	RBMS1	SPEN	UBTF	
ALG13	CIP29	DNAJC7	GNG12	KIF1A	MTHFD1L	PIP	RCL1	SPOP	UFL1	
ALKBH5	CKAP2	DNM2	GOT2	KIF23	MTHFD2	PITPNC1	RCOR1	SPTBN1	UHRF1	
AMOTL1	CKAP4	DNMBP	GPBP1	KIFC1	MYCBP	PKP2	RDH11	SPTBN2	UMPS	
ANLN	CKMT2	DOCK7	GPRC5A	KLF16	MYL1	PKP4	RECQL	SPTLC1	UNC45A	
ANP32A	CLPB	DPF2	GPX3	KLF4	MYL6B	PLAT	REXO4	SQSTM1	URB1	
ANP32E	CLPX	DPYSL2	GPX4	KPNB1	MYO15A	PLEKHA7	RFC2	SRBD1	USP39	
AP2A1	CLTC	DPYSL5	GRSF1	KRT78	MYO18A	PLEKHG	RFC3	SRM	USP5	
AP2B1	CMSS1	DRG1	GSN	KRTCAP2	MYO1B	PLRG1	RHOA	SRPK1	USP7	
AP4E1	CNIH4	DSG1	GSTM3	LA04NC01-25	MYO1D	PLS3	RING1	SRPK2	USP9X	
APC	CNN2	DSG2	GTF3C3	LANCL2	MYO1E	POLR1C	RIOK1	SRRM1	UTP23	
APEX1	CNP	DUSP11	GTF3C4	LARP4B	MYO6	POLR2B	RNF114	SSFA2	UTP3	
APOBEC3A	COA5	DVL2	GTF3C5	LARS	NAA10	PON2	RNF185	SSR1	UTP6	
APOBEC3C	COBL	DYNC1H1	GTPBP10	LAS1L	NACA	PPAN-P2	RNF2	STAU2	UTRN	
APRT	COPA	DYNLL2	HABP2	LBR	NAP1L1	PPIL1	RNPEP	STK26	V5-4	
ARF1	COPB1	EARS2	hCG_1821276	LIMA1	NARG1	PPIL4	ROCK1	SUCLG1	VAPA	
ARF4	COPB2	ECEL1	hCG_1989366	LIMCH1	NCAPG	PPP1R12	RPH3AL	SUPT5H	VAPB	
ARHG	COPG1	ECHS1	hCG_1996054	LIN7C	NCAPH	PPP1R13	RPL39P5	SURF4	VARS	
ARHGAP17	COX3	EFHD1	hCG_2024613	LOC392647	NCBP1	PPP1R9A	RPL7L1	SVIL	VASP	
ARHGEF1	CP	EGFR	hCG_2032701	LOC84524	NCLN	PPP1R9B	RPRD2	SYNM	VDAC1	
ARPC1B	CPNE3	EIF2A	HCTP4	LPCAT1	NDNL2	PPP2CA	RPS28	SYNPO	VDAC3	
ARPC2	CPSF2	EIF2B2	HDAC1	LRRC1	NDUFA10	PPP2R1A	RRAS2	TACO1	VDP	
ARPC3	CPSF3	EIF2C2	HEL-S-1	LRRC47	NDUFA13	PPP2R2A	RRP36	TAF10	VEZF1	
ASNS	CPT1A	EIF2S1	HEL-S-100n	LRRFIP1	NDUFA5	PRC1	RSBN1L	TBC1D10	VKORC1	
ATL3	crn	EIF3A	HEL-S-108	LRRFIP2	NDUFB5	PRIC295	S	TBL3	VL1	
ATP2B1	CSE1L	EIF3C	HEL-S-128m	LSG1	NDUFS1	PRKAR2	S100A10	TCEB2	VPS35	
ATP5F1	CSNK2A1	EIF3F	HEL-S-22	LTV1	NDUFS5	PRKCA	S100A16	TCERG1	VPS4A	
ATP6V1G2-D	CSNK2B	EIF3G	HEL-S-269	LUZP1	NELFE	PRKCI	S100A6	TECR	WDR12	
ATP6V1H	CSTF1	EIF3L	HEL-S-273	LYN	NEXN	PRKRA	SAMHD1	TES	WDR18	
AURKB	CTBP1	EIF4G2	HEL-S-29	MACF1	NF2	PRPF38B	SART3	TFB1M	WDR3	
BAF53A	CTNNA1	ELMSAN1	HEL-S-2a	MAGOHB	NFX1	PRPF4B	SCIN	TFRC	WDR36	
BAIAP2L1	CTNNB1	ELOF1	HEL-S-39	MAGT1	NIP7	PRPS1	SCRIB	THBS1	WDR43	
BAZ2A	CTNND1	EPB41L1	HEL-S-62p	MAP2K2	NIPSNAP1	PSIP1	SDAD1	THOC2	WDR5	
BCAS2	CTPS1	EPB41L2	HEL-S-66p	MAP2K3	NKAP	PSMA5	SEC24C	THOC5	WDR6	
BCKDHA	CXorf57	ERICH3	HEL-S-68p	MAP7D3	NMD3	PSMC4	SEC61A1	TIMM23B	WDR75	
BOD1L1	CYB5R3	ERLIN2	HEL-S-70p	MAP9	NME1	PSMC5	SEC61B	TIMM50	WRNIP1	
BPTF	CYC1	ERP44	HEL-S-73	MARCKSL1	NME4	PSMD1	SEH1L	TJP1	XPOT	
BRD4	CYTSA	ERP70	HEL-S-77p	MARS	NOL10	PSMD12	SENP3	TJP2	YAP1	
BSG	DAP3	ESYT2	HEL-S-87p	MAT2A	NOL11	PSMD13	SEP--2	TLE3	YARS	
C10orf47	DAPK3	ETHE1	HIGD1A	MCM2	NOL6	PSMD14	SERF2	TMED10	YTHDF1	
C12orf10	DARS2	EXOSC3	HJURP	MDC1	NPEPPS	PSMD2	SF3A1	TMEM109	ZBTB11	
C1orf33	DCTN4	EXOSC5	HM13	MECP2	NPM3	PSMD4	SF3A3	TMEM113	ZC3H15	
C1orf57	DDOST	EXOSC9	HMGA1	MFAP1	NQO1	PSMD6	SF3B3	TMEM165	ZCCHC10	
C7	DDX10	EZH2	HNRNPDL	MGC3731	NSF	PSMG1	SFRS11	TMEM200	ZCCHC3	
C7orf11	DDX19A	F5	HOMER1	MGST3	NUP107	PTDSS1	SFRS17A	TMEM214	ZFYVE19	
C8orf33	DDX24	FAM208A	HS24/p52	MISP	NUP133	PTGES3	SFXN3	TMEM63A	ZGRF1	
CAMK2D	DDX31	FAM208B	HSD17B10	MLLT4	NUP160	PTK2	SFXN4	TMOD1	ZMPSTE2	
CAMK2G	DDX49	FAM53C	HSD17B4	MPDZ	NUP188	PTPN1	SH3GL1	TMOD2	ZMYND8	
CAPZA2	DDX51	FAM76A	HSP90AB2P	MPG	NUP205	PTRF	SIPA1	TNKS1BP	ZNF185	
CAPZB	DDX56	FAM76B	HSPA14	MPP5	NUP43	PUF60	SIPA1L1	TNPO1	ZNF22	
CASC3	DECR2	FARSB	HUWE1	MPRIP	NUP62	PUM1	SIPA1L3	TOE1	ZNF281	
CAT56	DGKK	FGF2	ICT1	MRPL16	NUP85	PUS7	SKP1	TOMM20	ZNF316	

Supplementary Table S7 Mass spectrometry analysis of proteins pulled down by Flag antibody

AAAS	C1ORF33	CTNND1	EIF3A	HCG 199605	LIMCH1	MYO1E	PIH1D1	RANBP1	SLC7A5	TNPO1	YTHDC2
AASDHPP	C1ORF57	CTPS1	EIF3M	HCG_202273	LIN7C	MYO5A	PIP	RANBP5	SLIRP	TOE1	ZAK
ABLIM1	C3ORF17	CTR9	EIF4E	HCG_202461	LOC392647	MYO6	PITPNC1	RANGAP1	SLMAP	TOMM20	ZBTB1
ABT1	C8ORF33	CXORF57	EIF4G2	HCG_2032701	LOC84524	MYOF	PKP2	RAP2C	SLU7	TOMM34	ZC3H15
ACADVL	CAMK2D	CXXC1	ELMSAN1	HCG_39854	LPP	NAA10	PKP4	RARS	SMARCD2	TOMM40	ZCCHC1
ACLY	CAMK2G	CYC1	EP400	HCG_41772	LRRC1	NACA	PLAT	RASL11B	SMARCE1	TOMM70A	ZCCHC3
ACOT9	CAPZA2	CYTSA	EPB41L1	HDAC1	LRRC47	NAP1L1	PLEC1	RBFOX1	SMC2	TOP3B	ZFC3H1
ACSL3	CAPZB	DAD1	EPB41L2	HDGFRP2	LRRFIP1	NAP1L4	PLEKHA7	RBM12B	SMC4	TP53BP2	ZFYVE1
ACTN1	CAT56	DAP3	ERGIC1	HEL-S-1	LRRFIP2	NARG1	PLEKHG3	RBM15B	SMC5	TPM1	ZGRF1
ACTN4	CBX4	DAPK3	ERICH3	HEL-S-100N	LUZP1	NCAPD2	PLRG1	RBM22	SMPD4	TPM3	ZMPSTE
ACTR1A	CBX6	DARS2	ERLIN2	HEL-S-106	LYN	NCAPG	PLS3	RBM26	SMU1	TPM4	ZNF185
ACTR2	CCDC12	DCAF7	ERP44	HEL-S-108	LZTS2	NCAPH	POGZ	RBMS1	SNRPB2	TPRN	ZNF22
ACTR3	CCDC168	DCTN4	ERP70	HEL-S-128M	MACF1	NCBP1	POLDIP2	RCC1	SNRPG	TPT1	ZNF280
ADH5	CCDC47	DDOST	ESYT2	HEL-S-19	MACROD1	NCKAP1	POLE3	RCL1	SNW1	TRAF4	ZNF316
AFAP1	CCDC86	DDX10	ETHE1	HEL-S-22	MAGOHB	NCLN	POLR1C	RCN2	SORBS3	TRIM21	ZNF346
AGBL5	CCT7	DDX20	EXOSC2	HEL-S-269	MAGT1	NDNL2	POLR2B	RCOR1	SP1	TRIM25	ZNF461
AGK	CCT8	DDX24	EXOSC3	HEL-S-273	MAP2K2	NDUFA10	POLRMT	RDH11	SPATA5	TRIM27	ZNF569
AGO2	CD2BP2	DDX31	EXOSC6	HEL-S-29	MAP2K3	NDUFA13	POM121C	RECQL	SPECC1	TRIM4	ZNF593
AGPS	CD59	DDX51	EZH2	HEL-S-2A	MAP7D3	NDUFA5	POP7	REXO4	SPEN	TRIOBP	ZNF622
AHCY	CDC2	DDX55	F5	HEL-S-62P	MARCKSL1	NDUFS1	PPAN-P2RY11	RFC2	SPTBN1	TRIP13	ZNF668
AHSG	CDC40	DDX56	FAM208B	HEL-S-66P	MARS	NDUFS5	PPIL1	RFC3	SPTBN2	TRIP6	ZNF771
AIF1L	CDC42EP1	DECR2	FAM76A	HEL-S-68P	MAT2A	NDUFS7	PPIL4	RHOA	SPTLC1	TRMT112	ZRANB2
AIMP2	CDK9	DERL1	FAM76B	HEL-S-73	MBD3	NDUFS8	PPP1R12A	RING1	SQSTM1	TSFM	
AKAP2	CECR5	DGKK	FAM83H	HEL-S-77P	MBNL1	NELFE	PPP1R13L	RIOK1	SRPK1	TSPO	
AKAP8L	CELF1	DHCR7	FARP1	HEXIM1	MCM2	NES	PPP1R9A	RNASEH1	SRPK2	TSR1	
ALDH18A1	CENPB	DHX29	FERMT2	HMGA1	MDC1	NEXN	PPP1R9B	RNF114	SSB	TSR3	
ALDH1B1	CENPV	DHX30	FGF2	HMMR	MECP2	NF2	PPP2CA	RNF185	SSFA2	TUFT1	
ALDH2	CEP63	DHX33	FKBP8	HNRNPDL	MEPCE	NFX1	PPP2R1A	RNF2	SSR1	TWF1	
ALDH7A1	CFI	DHX57	FLII	HOMER1	MFAP1	NIP7	PPP2R2A	RNMTL1	SSR3	TXLNA	
ALDH9A1	CGN	DHX8	FLJ00163	HS24/P52	MGC3731	NMD3	PPP3CA	RPA1	SSR4	UACA	
ALG1	CHAMP1	DIS3	FLJ00385	HSD17B10	MISP	NME4	PRC1	RPH3AL	STAU2	UBAP2	
ALPL	CHCHD3	DKFZP434E1119	FLJ10154	HSD17B4	MLF2	NOB1	PRIC295	RPL7L1	STIP1	UBE1	
AMOTL1	CHMP1A	DKFZP586K0821	FN1	HSP90AB2P	MLLT4	NOC2L	PRKAR2A	RPP30	STK26	UBTF	
ANLN	CHMP2A	DKFZP586O0821	FUBP1	HSPA14	MPDZ	NOL10	PRKCA	RPS28	STOML2	UBXN1	
ANP32A	CHMP4B	DKFZP686A111	GADD45GIP1	HSPH1	MPG	NOL11	PRKCI	RRAS2	SUCLG1	UCHL5	
ANP32E	CHORDC1	DKFZP686E1899	GALE	HUWE1	MPP5	NOL6	PRPF3	RRP36	SUGP2	UCK2	
AP2A1	CIAO1	DKFZP686E2327	GAN	HYOU1	MPRIP	NOL8	PRPF31	RSBN1	SUPT5H	UFL1	
AP2B1	CIP29	DKFZP686G2045	GAPVD1	IARS2	MRPL16	NOP10	PRPF38B	S	SURF4	UMPS	
AP2M1	CKAP2	DKFZP686J0330	GART	IDH3B	MRPL19	NPM3	PRPF4B	S100A10	SURF6	UNC45A	
AP4E1	CKAP4	DKFZP686J162	GBX2	IGF2	MRPL22	NSF	PRPS1	S100A16	SVIL	URB1	
APC	CKAP5	DKFZP686L0869	GEMIN4	IGKV1-8	MRPL24	NUP107	PSIP1	S100A6	SYNM	URCC5	
APEX1	CKMT2	DKFZP686M1669	GFM1	IKBKAP	MRPL3	NUP133	PSMA4	S100P	SYNPO	USP14	
APOBEC3	CLOCK	DKFZP762C1015	GFPT1	ILK	MRPL38	NUP160	PSMA5	SAE1	TACO1	USP5	
APRT	CLPB	DKFZP762I166	GJA1	ILKAP	MRPL4	NUP188	PSMC4	SAMM50	TBC1D10B	USP7	
ARCN1	CLPX	DKFZP779I1858	GLE1	IMP3	MRPL40	NUP205	PSMC5	SART3	TBL1XR1	USP9X	
ARF1	CLTC	DMD	GLRX3	IMP4	MRPL41	NUP43	PSMD1	SCAMP3	TBL3	UTP23	
ARF4	CNIH4	DNAJB1	GLYR1	INADL	MRPL47	NUP85	PSMD12	SCIN	TCEB2	UTP3	
ARHG	CNN2	DNAJB11	GMPS	IPO7	MRPL48	NUP88	PSMD13	SCML2	TECR	UTP6	
ARHGAP1	CNOT1	DNAJB12	GNA11	IRS4	MRPL49	NVL	PSMD14	SCO2	TES	UTRN	
ARHGEF1	CNP	DNAJB6	GNA13	ITPR3	MRPL54	NXT2	PSMD2	SCRIB	TEX10	VAPA	
ARL6IP4	COA5	DNAJC11	GNAI2	JUP	MRPL55	OAS3	PSMD4	SDAD1	TFAM	VAPB	
ARPC1B	COBL	DNAJC2	GNAI3	KANK2	MRPS10	OGT	PSMD6	SEC23A	TFB1M	VARS	
ARPC3	COPA	DNAJC7	GNAS	KDELR2	MRPS12	OSTC	PSMD8	SEC24C	TFCP2	VASP	
ARPC4	COPB1	DNM2	GNB1	KHDRBS3	MRPS15	OXSR1	PSMG1	SEC61A1	TFRC	VDAC1	
ASNS	COPB2	DNMBP	GNB2	KIAA0020	MRPS16	PA2G4	PTCD3	SEC61B	THBS1	VDAC3	
ATL3	COPE	DOCK7	GNB4	KIAA0992	MRPS23	PACSIN3	PTDSS1	SENP3	THEX1	VDP	
ATP2B1	COPG1	DPF2	GNG12	KIAA1671	MRPS25	PAF1	PTPN1	SEP-2	THOC2	VEZF1	
ATP5F1	CORO2A	DPYSL2	GOLGA2	KIF14	MRPS26	PAICS	PUF60	SEP-7	THOC3	VPS18	
ATP5H	COX3	DRG1	GOT2	KIF22	MRPS28	PARD3	PUM1	SEP-9	THOC5	VPS35	
ATP5J2-PT	COX7A2	DSG1	GPBP1	KIFC1	MRPS31	PBRM1	PUS7	SERF2	TIMM50	VPS37B	
ATP6V1G2	CP	DSG2	GPR50	KLF16	MRPS6	PCK2	PUSL1	SF3A1	TJP1	VPS4A	
ATRN	CPNE1	DVL2	GPRC5A	KLF4	MSH2	PCM1	PYCR1	SF3B3	TJP2	WDR12	
AURKB	CPNE3	DYNC1H1	GPX3	KPNB1	MSH6	PDCD4	PYCR2	SFRS17A	TLN1	WDR18	
BAF53A	CPSF2	DYNC1LI1	GPX4	KRT78	MST065	PDCD6IP	PYCRL	SH3GL1	TM9SF3	WDR3	
BAIAP2L1	CPSF3	DYNLL2	GRSF1	KRTAP2-1	MTA1	PDIA3	PYGL	SIPA1L1	TMED10	WDR36	
BAZ2A	CPT1A	EARS2	GSN	KRTCAP2	MTCH2	PDK1	RAB11A	SIPA1L3	TMEM109	WDR43	
BCAS2	CRIP1	EED	GSPT1	LA04NC01-25	MTDH	PDLIM7	RAB11FIP1	SKP1	TMEM11	WDR5	
BCOR	CRN	EFHD1	GTF3C3	LAMB1	MTHFD1L	PDS5B	RAB14	SLC16A1	TMEM113	WDR6	
BPTF	CS	EGFR	GTF3C4	LANCL2	MTHFD2	PFKL	RAB1A	SLC16A3	TMEM165	WDR75	
BRD2	CSE1L	EHD1	GTF3C5	LARP4B	MYCBP	PFN1	RAB1B	SLC25A1	TMEM205	XPO1	
BRD4	CSNK2A1	EHD4	GTPBP1	LARS	MYL1	PFN2	RAB34	SLC25A10	TMEM214	XPO5	
BSG	CSNK2B	EIF2A	HABP2	LAS1L	MYL6B	PGAM1	RAB7A	SLC25A11	TMEM263	XPOT	
C10ORF47	CTBP1	EIF2B2	HAT1	LEMD3	MYO18A	PHF3	RAB9A	SLC25A13	TMOD1	YAP1	
C18ORF21	CTNNA1	EIF2D	HCG_182127	LIG3	MYO1B	PHGDH	RAI14	SLC2A1	TMOD2	YARS	
C19ORF70	CTNNB1	EIF2S1	HCG_198936	LIMA1	MYO1D	PHIP	RALB	SLC39A7	TNKS1BP1	YME1L1	
